# microRNA‐425 loss mediates amyloid plaque microenvironment heterogeneity and promotes neurodegenerative pathologies

**DOI:** 10.1111/acel.13454

**Published:** 2021-09-12

**Authors:** Yong‐Bo Hu, Yong‐Fang Zhang, Ru‐Jing Ren, Eric B. Dammer, Xin‐Yi Xie, Shi‐Wu Chen, Qiang Huang, Wan‐Ying Huang, Rui Zhang, Hong‐Zhuan Chen, Hao Wang, Gang Wang

**Affiliations:** ^1^ Department of Neurology and Neuroscience Institute Ruijin Hospital Affiliated to Shanghai Jiao Tong University School of Medicine Shanghai China; ^2^ Department of Pharmacology and Chemical Biology Shanghai Jiao Tong University School of Medicine Shanghai China; ^3^ Department of Neurology,Shanghai East Hospital School of Medicine,Tongji University Shanghai China; ^4^ Department of Biochemistry and Center for Neurodegenerative Disease Emory University School of Medicine Atlanta Georgia USA; ^5^ Institute of Interdisciplinary Science Shuguang Hospital Shanghai University of Traditional Chinese Medicine Shanghai China

**Keywords:** Alzheimer's disease, amyloid plaque, microenvironment, miR‐425, neurodegeneration, oligonucleotide

## Abstract

Different cellular and molecular changes underlie the pathogenesis of Alzheimer's disease (AD). Among these, neuron‐specific dysregulation is a necessary event for accumulation of classic pathologies including amyloid plaques. Here, we show that AD‐associated pathophysiology including neuronal cell death, inflammatory signaling, and endolysosomal dysfunction is spatially colocalized to amyloid plaques in regions with abnormal microRNA‐425 (miR‐425) levels and this change leads to focal brain microenvironment heterogeneity, that is, an amyloid plaque‐associated microenvironment (APAM). APAM consists of multiple specific neurodegenerative signature pathologies associated with senile plaques that contribute to the heterogeneity and complexity of AD. Remarkably, miR‐425, a neuronal‐specific regulator decreased in AD brain, maintains a normal spatial transcriptome within brain neurons. We tested the hypothesis that miR‐425 loss correlates with enhanced levels of mRNA targets downstream, supporting APAM and AD progression. A miR‐425‐deficient mouse model has enhanced APP amyloidogenic processing, neuroinflammation, neuron loss, and cognitive impairment. In the APP/PS1 mouse model, intervening with miR‐425 supplementation ameliorated APAM changes and memory deficits. This study reveals a novel mechanism of dysregulation of spatial transcriptomic changes in AD brain, identifying a probable neuronal‐specific microRNA regulator capable of staving off amyloid pathogenesis. Moreover, our findings provide new insights for developing AD treatment strategies with miRNA oligonucleotide(s).

## Graphical abstract

The present study demonstrates that miR‐425 loss is responsible for PI3K‐Akt signaling suppression and impaired NPC proliferation. Moreover, miR‐425 loss induces dendritic spine defects and impaired synaptic plasticity. Taken together, miR‐425 loss following initial plaque deposition is the opening of the floodgates that mediates the ability of the APAM to cascade into a cellular phase of AD.

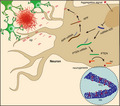

## 1 INTRODUCTION

Alzheimer's disease (AD) is an age‐related dementia with progressive cognitive impairment (Hampel et al., 2018). This devastating neurological disease is characterized histopathologically by the extracellular aggregation of amyloid plaques, intraneuronal neurofibrillary tangles (NFTs), activated neuroinflammation, and neuron loss (Shi et al., 2018; Xia et al., 2018). These alterations comprise a framework of our understanding of AD pathology. Although disparate cell types and molecular dysregulation events are involved in the pathogenesis of AD (Fakhoury, 2018), there are likely to be neuronal‐specific dysregulation events that link the pathologies centering on neuronal processes, that is, both the appearance of NFTs, and the amyloidogenic processing of amyloid precursor protein (APP) which precedes NFTs appearance. Therefore, we hypothesize specific regulator(s) are a link between the complex pathologies in our current framework of understanding AD, and we prefer to investigate them, rather than potential drivers of a single pathology.

The amyloid hypothesis has provided a framework for investigating AD pathogenesis, wherein the extracellular polymerization and aggregation of amyloid‐β peptide (Aβ) triggers a cascade of pathologic events that leads to neuroinflammation, cell death, and neurodegeneration that ultimately leads to dementia (Henstridge et al., 2019). Extracellular amyloid plaques display a highly complex structure in which aggregated Aβ peptides are entangled with glial cells and neuronal components (Kreutzer & Nowick, 2018); this comprises a pathological microenvironment. Many proteins colocalize or co‐isolate with amyloid plaques, as identified by different biochemical approaches including immunohistochemical (IHC) staining, LC‐MS/MS, and transcriptomic analysis (Castanho et al., 2020; Han et al., 2016). These proteins are involved in a variety of cellular functions including proteolysis, membrane trafficking, inflammation, cell adhesion, metabolism, and cytoskeleton assembly. These findings support the existence of a complex network of biochemical changes across cell types occurring in conjunction with amyloid aggregation (Hussain et al., 2019). Perhaps less appreciated is that this network of changes across cells is occurring in an amyloid plaque‐associated microenvironment (APAM), which consists of multiple specific neurodegenerative pathologies associated with senile plaques that contribute to the heterogeneity and complexity of AD (Prokop et al., 2019).

In the brain regions with observed amyloid aggregation, multiple specific neurodegenerative pathologies are usually co‐observed in close proximity, though appearing in a predictable sequence over time, which suggests a critical early role of amyloid aggregation intimating interactions with other neurodegenerative pathologies (Long & Holtzman, 2019; Pereira et al., 2019). For example, NFTs with hyperphosphorylated tau are induced by Aβ oligomers from AD brains and accumulated Aβ in vivo may initiate the hyperphosphorylation of tau (De Felice et al., 2008). Moreover, several inflammation‐associated proteins such as HTRA1 and complement C1q and C3 are found to colocalize with amyloid plaques in AD brain, suggesting that aberrant activation of microglia may contribute to neurodegeneration and synaptic dysfunction (Fonseca et al., 2004). APP and BACE1 are also colocalized and enriched in the vicinity of amyloid plaques, suggestive of feed‐forward by amyloid plaques to accelerate production and subsequent spread of Aβ (Zhao et al., 2007). Moreover, U1 small nuclear RNA (snRNA) and snRNP subunits are also found to be colocalized with Aβ aggregation (Hales et al., 2014). These components of spliceosomes are responsible for mRNA splicing, suggesting that post‐transcriptional dysregulation is also involved in amyloid plaque‐mediated pathologies (Millan, 2017).

MicroRNA (miRNA) is an important post‐transcriptional regulator also involved in neurodegeneration. miRs are small (~18–24 nt) noncoding RNAs that bind predominantly to the 3′ untranslated regions of target mRNAs and regulate their expression. Many miRNAs are highly conserved across species and regulate important physiological and pathophysiological signaling pathways (Swarup et al., 2019). Several miRNAs are identified to be associated with aspects of AD pathogenesis including Aβ production, tau phosphorylation, inflammation, and cell death (Juzwik et al., 2019; Williams et al., 2017). Some aspects of the above miRNA‐associated processes are integrated into a framework for understanding the complexity of the aged, often increasingly heterogeneous, microenvironment to promote dyshomeostasis and disease, and the idea of permissive microenvironment is particularly developed in the cancer field, where much work has been performed to elucidate tumor microenvironment (TME) and tumor immune microenvironment (TIME) (Ohandjo et al., 2019). Brain tissue microenvironment affecting permissiveness for AD progression is rarely appreciated, and importantly, no study has yet investigated the role of miRNAs in the molecular networks of APAM during pathological AD progression.

In the present study, we tested the hypothesis that miR‐425 prevents APAM by suppressing the expression of factors supporting a localized microenvironment conducive to AD progression. First, we confirmed that the focal brain heterogeneity of APAM arised from post‐transcriptional dysregulation, resulting in a cascade of pathological changes associated with AD. We identified a specific neuronal regulator miR‐425 through spatial transcriptomics as a key suppressor of APAM changes that are upstream of neurodegeneration. Furthermore, we developed a mouse model of miR‐425 deficiency which mimics amyloid plaque‐associated microenvironmental changes and found that miR‐425 loss leads to neurodegeneration and cognitive impairment in this mouse, which has pathological phenotypes very similar to AD. Finally, we showed that miRNA mimics AgomiR‐425 oligonucleotide treatment ameliorates pathological and behavior phenotypes in the APP/PS1 AD model, suggesting that modifying brain microenvironment by supplementation of miR‐425 could be a therapeutic strategy for treating AD.

## 2 RESULTS

### 2.1 Amyloid aggregation induces spatial transcriptomic changes in the brain of an AD mouse model

**FIGURE 1:**
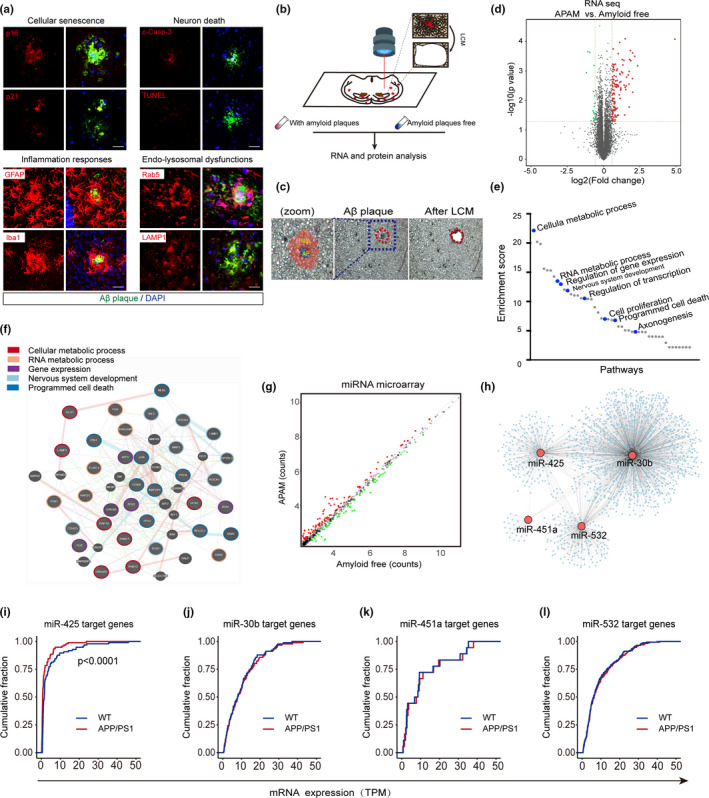
Amyloid aggregation induces spatial transcriptomic changes in the brain of APP/PS mice. (a) Colocalization of cellular senescence, neuron death, inflammation, and endosomal–lysosomal dysfunctions with amyloid plaques. Scale bars, 50 μm. (b) Schematic illustration of laser capture microdissection (LCM) and RNA sequencing. (c) Representative Aβ (NAB228) stained brain section for LCM. AP, amyloid plaques. Scale bars, 100 μm. (d) RNA‐seq volcano plot of captured APAM and amyloid‐free regions. (e) Gene ontology (GO) enrichment and network analysis of RNA‐seq. (f) Dysregulated mRNA and miRNA co‐expression network in APAM. (g) miRNA microarray volcano plot of captured APAM and amyloid‐free regions. (h) Differentially expressed miRNAs and their potential target genes in miRNA seq. (i–l) Cumulative distributions of target mRNAs expressions for miR‐425, miR‐30b, miR‐451, miR‐532 between WT and APP/PS1 mice. *p* Values were calculated by two‐sided Kolmogorov–Smirnov (K‐S) test

To reveal complex AD pathological changes localized to amyloid plaques, we performed immunofluorescence on brain slices of 15‐month‐old APP/PS1 mice. Compared to regions without aggregated amyloid, markers of cellular senescence (p16, p21), neuronal death (cleaved Casp3, TUNEL), inflammation (GFAP, Iba1), and endosomal–lysosomal dysfunction (Rab5, LAMP1) were all colocalized with amyloid plaques (Figure 1a, Figure S1 A‐D). These pathological marks fit within a complex pathological framework of understanding neurodegeneration downstream mainly of amyloid aggregation (Hussain et al., 2019). Here, we pose the usefulness of regarding that framework as a feature of the aged tissue, coining the term of amyloid plaque‐associated microenvironment (APAM). This framework arises the hypothesis that focal amyloid aggregation induces brain microenvironment heterogeneity mediating cascade(s) of molecular dysregulation that lead to overt neurodegeneration. The heterogeneity of tissue giving rise to APAM may result from changes in spatial transcriptomics in preclinical AD brain (Figure S2A–C). To precisely investigate the changes in spatial transcriptome deriving from amyloid aggregation, we used laser‐capture microdissection (LCM) to capture brain microenvironment affected by proximal amyloid plaques with distal amyloid plaque‐free regions of APP/PS1 mice, and compared mRNA expression by RNA sequencing (Figure 1b,c). We identified 504 genes significantly altered in APAM compared with amyloid plaque‐free regions (Figure 1d). Gene ontology (GO) analysis of differentially expressed genes (DEGs) revealed that they were involved in cellular metabolic process(es), RNA processing, gene expression, nervous system development, and programmed cell death. In particular, the enrichment of gene products with roles in RNA metabolism, gene expression, and transcriptional dysregulation prompted us to further explore the probable role of miRNAs in APAM (Figure 1e,f). miRNA microarray analysis demonstrated the up‐regulation of 95 miRNAs and downregulation of 64 miRNAs in APAM relative to amyloid plaque‐free regions (Figure 1g). To further identify the alterations of spatial expression profiles, we generated a mRNA‐miRNA co‐expression network, and a total of 4 interconnected subnetworks of target nodes potentially downstream of dysregulated miRNAs were obtained, including miR‐425, miR‐451a, miR‐532, and miR‐30b (Figure 1h). To further explore the role of these miRNAs in amyloid pathology, we predicted potential targets of miRNAs and referenced RNA sequencing data of APP/PS1 hippocampus. The results showed that target genes of miR‐425 were more impacted in the brain of APP/PS1 mice, suggesting that miR‐425 may exert a global impact on gene expression involved in AD‐like pathogenesis in APP/PS1 mice (Figure 1i–l). Additionally, miR‐425 was also significantly reduced in the hippocampus of APP/PS1 mice (Figure S2D). Consistent with these observations, previous studies also found a marked reduction of miR‐425 level in the brain of AD patients through different methods including RNA‐seq and RT‐PCR (Cogswell et al., 2008; Lau et al., 2013). Moreover, in our prior diagnostic screening trials with peripheral blood, miR‐425 was also found significantly dysregulated in AD patients relative to healthy controls (Ren et al., 2016). Therefore, we focused the remainder of our study on the role of miR‐425 as a specific neuronal regulator influencing APAM changes in AD pathogenesis.

### 2.2 miR‐425 is decreased in the AD brain

**FIGURE 2:**
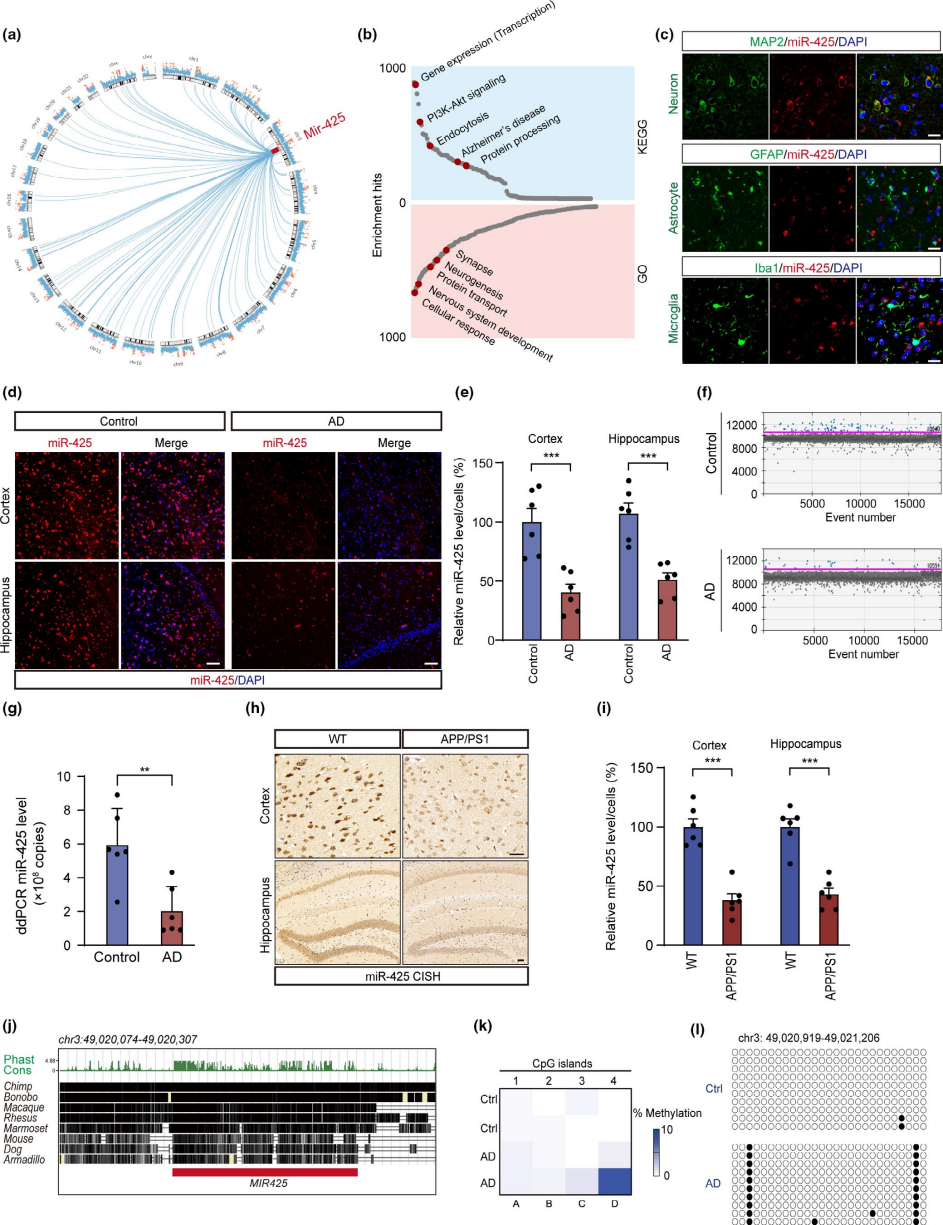
miR‐425 is decreased in the AD brain. (a) CIRCOS plot showed Mir425 (red) and its target genes. (b) KEGG and GO enrichment of miR‐425 predicted target genes. (c) miR‐425 (red) was localized with MAP2‐positive neurons not with GFAP/Iba1‐positive glial cells. Scale bars, 20 μm. (d, e) In situ hybridization and quantification of miR‐425 in hippocampus and cortex of AD patients, *n* = 6. Scale bars, 50 μm. (f, g) Representative droplet digital PCR (ddPCR) and quantification of miR‐425 in hippocampus of AD patients. (h, i) In situ hybridization and quantification of miR‐425 in hippocampus and cortex of 15‐month‐old APP/PS1 mice, *n* = 6. Scale bars, 50 μm. (j) Conservation analysis of Mir425 gene in different species. (k, l) The heatmap and methylation levels in promoter regions of Mir425 gene detected by bisulfite sequencing PCR in the hippocampus of AD patients. Data were presented as mean ± SEM. Two‐tailed unpaired Student's *t* test. **p < 0.01, ***p < 0.001

To elucidate the potential role of miR‐425 in AD, we identified miR‐425 target genes with target prediction tools (Targetscan, Tarbase, and miRbase) (Figure 2a). A detailed analysis of the predicted target genes revealed that miR‐425 could be implicated in transcription, PI3K‐Akt signaling, endocytosis, AD, and protein processing (Figure 2b). Gene Ontology (GO) analysis indicated regulatory effects of miR‐425 on cellular response(s), nervous system development, protein transport, neurogenesis, and synapses. As the brain milieu encompasses multiple cell types, we explored which cell types express miR‐425 and found that miR‐425 was most enriched in neurons, in line with target gene enrichment analysis (Figure 2c, Figure S3A–C). To further confirm the expression of miR‐425 in AD, we performed in situ hybridization of miR‐425. The results showed that the level of miR‐425 was greatly decreased in the hippocampus and cortex of AD patients and 15‐month‐old APP/PS1 mice (Figure 2d–i). Further evidence supporting miR‐425 loss in the hippocampus of AD patients was obtained using digital droplet PCR (Figure 2f,g). Importantly, we found that the level of miR‐425 expression was negatively correlated with Aβ aggregation in various brain regions of AD (Figure S3D,E), which suggested that miR‐425 may be associated with negative regulation of APP processing and amyloid production. Therefore, we further explored the mechanism of miR‐425 downregulation in AD.

Previous studies have confirmed that the methylation of genomic DNA affecting transcriptional machinery access was implicated in decreased miRNA gene expression (Song et al., 2019). To this end, we performed bisulfite sequencing PCR to detect the methylation levels of CpG islands in the promoter region of miR‐425 gene. Results confirmed a higher methylation of the miR‐425 gene promoter in the hippocampus of AD patients, with reduced miR‐425 expression, strengthening a hypothesis that miR‐425 may suppress APAM required for AD pathology (Figure 2k,l). Notably, the gene sequence of miR‐425 was conserved across species from mouse to human, indicating the consistency of its functions (Figure 2j).

**FIGURE 3:**
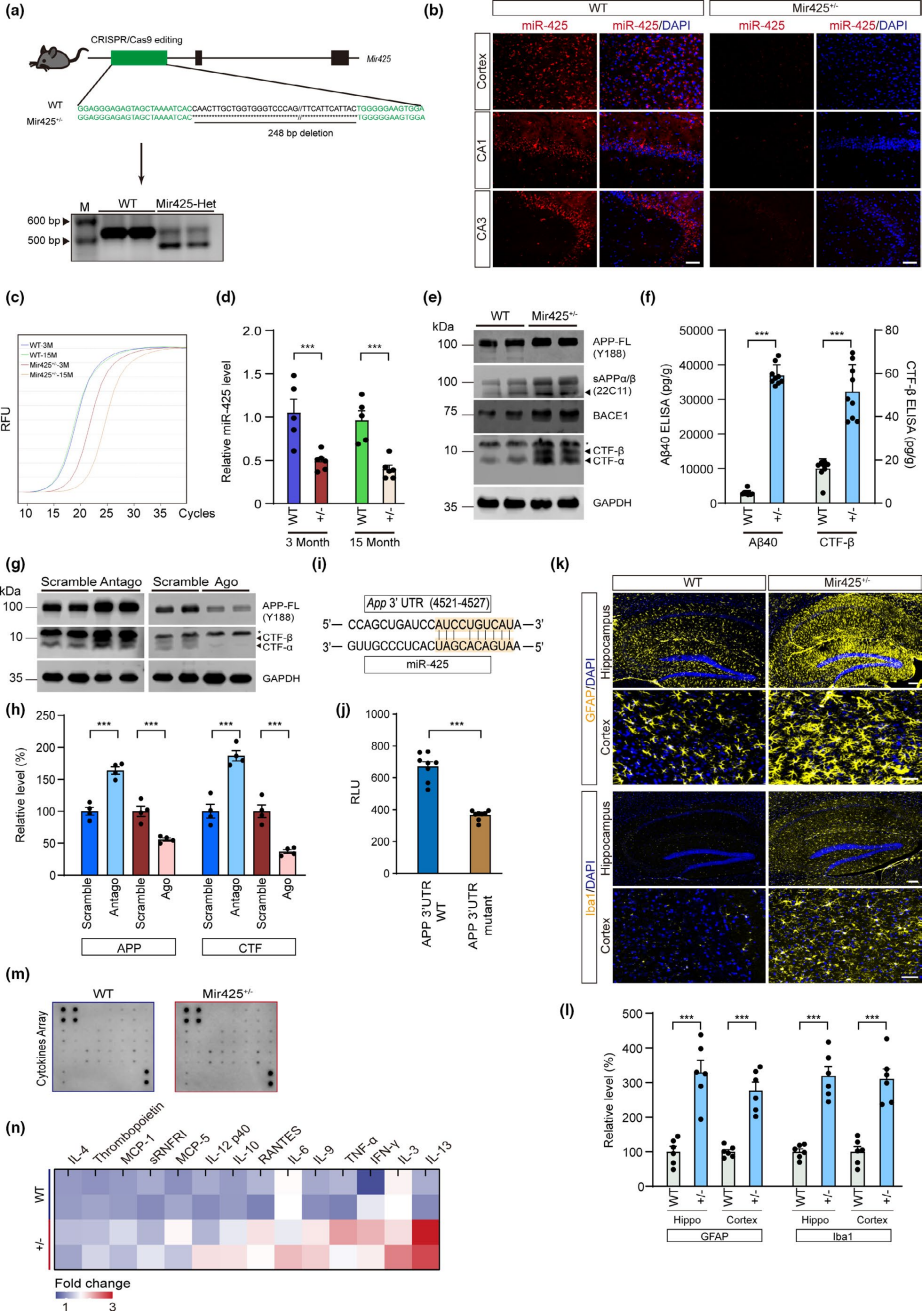
miR‐425 deficiency activates amyloidogenic APP processing and systematic neuroinflammation. (a) Generation of Mir425^+/−^ mice with CRISPR/Cas9 and genotypes verification of Mir425^+/−^ and WT mice using PCR. (b) In situ hybridization of miR‐425 in hippocampus and cortex of Mir425^+/−^ and WT mice. Scale bars, 50 μm. (c, d) RT‐PCR of miR‐425 level in the hippocampus of Mir425^+/−^ and WT mice at 3 months and 15 month. (e) Western blot of APP, BACE1, sAPPα/β, CTF‐α, and CTF‐β in the brain of miR‐425^+/−^ and WT mice. (f) ELISA of Aβ40 and CTF‐β in the brain of 15‐month‐old Mir425^+/−^ and WT mice. (g, h) Western blot of APP and CTF‐β in PC12 cells transfected with Antagomir‐425 and Agomir‐425. (i, j) Luciferase assay of miR‐425 and APP 3′UTR. (k, l) Immunofluorescence and quantification of GFAP‐positive and Iba1‐positive glial cells in the hippocampus of 15‐month‐old Mir425^+/−^ and WT mice. Scale bars, 50 μm. (m, n) Microarray cytokines assay of hippocampal cell lysates in 15‐month‐old Mir425^+/−^ and WT mice. Data were presented as mean ± SEM. Two‐tailed unpaired Student's *t* test. ***p < 0.001; **p < 0.01; *p < 0.05

To further assess the contribution of miR‐425 to APAM regulation and to potentially mimic pathological changes of APAM in vivo, we generated miR‐425‐deficient mice using CRISPR/Cas9 editing in C57BL/6 mice (Figure 3a). In the process of heterozygote intercross, we found that homozygosity for the Mir425 deletion was lethal in mice. However, Mir425 heterozygotes (Mir425^+/−^) did not display significant abnormalities nor did they have overall decreased survival compared to wildtype (WT) mice. Moreover, using a fluorescent probe detecting mature miR‐425, we observed that miR‐425 expression was deficient in the cortex and hippocampus of Mir425^+/−^ mice compared to WT (Figure 3b). miR‐425 deficiency was also confirmed using RT‐PCR (Figure 3c,d). Therefore, we used Mir425^+/−^ mice as a mimic of miR‐425 deficiency in the human AD brain.

### 2.3 miR‐425 deficiency activates amyloidogenic APP processing and increases reactive gliosis

To examine whether miR‐425 deficiency would lead to AD‐associated biochemical abnormalities, we first examined APP processing and Aβ generation in the hippocampus of 15‐month‐old miR‐425^+/−^ mice. Western blot results revealed that the expression of APP and BACE1 was increased (Figure 3e). ELISA results showed that soluble Aβ40 and CTF‐β were significantly increased relative to WT (Figure 3f). Immunofluorescence of APP full length, and Thioflavin S (ThS)‐positive Aβ plaque staining further confirmed that miR‐425 deficiency promoted the elevation of APP and Aβ production in neurons (Figure S4). Brain RNA analysis using qRT‐PCR revealed that the APP and BACE1 transcripts were also significantly up‐regulated in Mir425^+/−^ mice (Figure S4B). To further identify a causal relationship between miR‐425 and APP amyloidogenic processing, we performed loss‐ and gain‐of‐function experiments using PC12 cells stably expressing human Swedish mutations in APP^695^. In these cells, miR‐425 knockdown using antagomiR‐425 promoted APP expression and CTF‐β production. In contrast, miR‐425 overexpression using miR‐425 transfection decreased APP level and amyloidogenic fragments (Figure 3g,h). Using computational analysis for miRNA‐target prediction, we identified APP as a novel miR‐425 target; in addition to BACE1, we previously reported (Ren et al., 2016) (Figure 3i). Luciferase reporter analysis revealed that the APP 3′ UTR was sufficient to confer miR‐425 regulation on APP mRNA. Importantly, when the APP 3′ UTR was mutated to prevent the binding of miR‐425, miR‐425 regulation of the construct was abolished (Figure 3j). Collectively, our current and prior results suggest that miR‐425 deficiency promotes APP amyloidogenic processing through derepression of full length APP protein, and downstream, Aβ production is enhanced, additionally to loss of coordinated miR‐425 post‐translational suppression of BACE1.

Appearing sequentially following overproduction and accumulation of Aβ, reactive gliosis is another pathological characteristic of AD (Sierksma et al., 2020). In the brain of 15‐month‐old Mir425^+/−^ mice, we observed that miR‐425 deficiency induced higher amounts of activation of microglia and astrocytes relative to age‐matched WT controls (Figure 3k,l). Furthermore, an assay of cytokines in brain lysates confirmed that many cytokines were increased in the brain of Mir425^+/−^ mice, including IL‐13, IFN‐γ, IL‐9, and TNFα (Figure 3m,n). These data indicate that miR‐425 deficiency promotes reactive gliosis and a cascade of inflammation in the brain of Mir425^+/−^ mice.

### 2.4 Decreased miR‐425 promotes neuron loss via suppression of the PI3K‐AKT pathway

**FIGURE 4:**
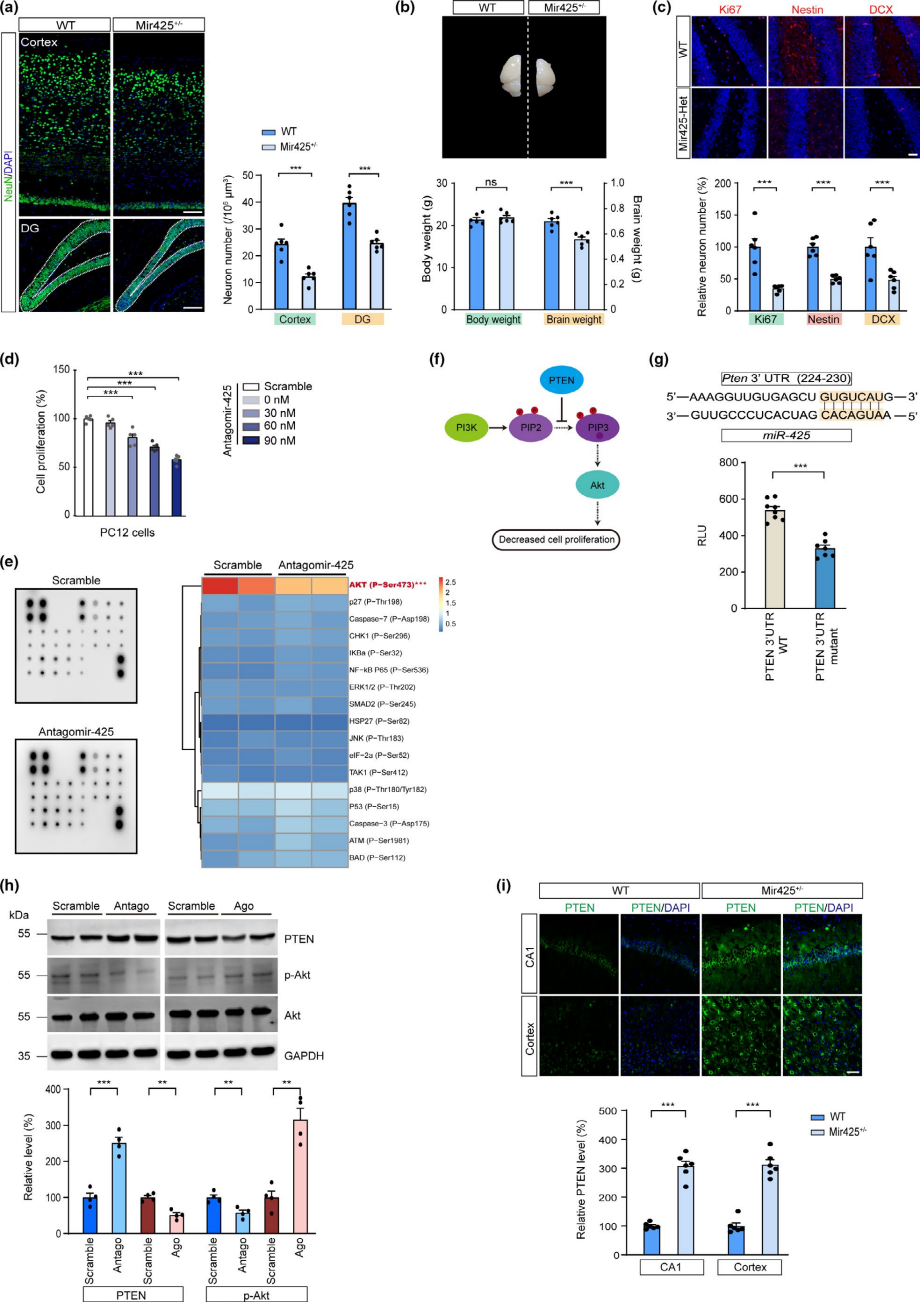
Decreased miR‐425 promotes neuron loss by suppressing the PI3K‐Akt pathway. (a) Immunofluorescence and quantification of NeuN in cortex and DG region of 15‐month‐old Mir425^+/−^ and WT mice. Scale bars, 100 μm. (b) Mouse brain and quantification of body and brain weight of Mir425^+/−^ and WT mice. (c) Immunofluorescence and quantification of Ki67‐, Nestin‐, and DCX‐positive neurons in hippocampus of Mir425^+/−^ and WT mice. Scale bars, 50 μm. (d) Cell proliferation analysis of PC12 cells transfected with different dosage of anatagomiR‐425. One‐way ANOVA followed by post hoc analysis. (e) Microarray assay of cell death and proliferation‐associated pathways in PC12 cells transfected with Antagomir‐425. (f) Schematic plot of PI3K‐Akt signaling pathway and PTEN. (g) Luciferase assay of miR‐425 and PTEN 3′UTR. (h) Western blot of PTEN, p‐Akt, and Akt in PC12 cells transfected with Antagomir‐425 and Agomir‐425. (i) Immunofluorescence and quantification of PTEN in the brain of Mir425^+/−^ and WT mice. Scale bars, 50 μm. Data were presented as mean ± SEM. Two‐tailed unpaired Student's *t* test. ***p < 0.001; *p < 0.05

Given that miR‐425 loss activated APP expression, and that previous studies reported APP regulated neurogenesis and enhanced neuronal progenitor cell differentiation during development (Zhang et al., 2014), we next investigated whether miR‐425 affects adult neurogenesis in mice. Immunofluorescence staining revealed a significant decrease in neurons number in the cortex and hippocampus of miR‐425‐deficient mice (Figure 4a). Consistently, we observed decreased brain weight in Mir425^+/−^ mice, with the body weight remaining unchanged (Figure 4b). Adult brain neurogenesis occurs through neuronal differentiation, cell proliferation, and positive regulation of cell survival (Pan et al., 2016). To test how neurogenesis was altered in miR‐425‐deficient brain, we used specific‐cell type marker labeling and found that Ki‐67 positive cells, and nestin‐ and DCX‐expressing immature neurons were markedly decreased in miR‐425‐deficient mice, which suggested that miR‐425 affected neuronal progenitor cell (NPC) proliferation and survival (Figure 4c). Meanwhile, we replicated this suppression of cell proliferation in a dose‐dependent manner in vitro with neuronal PC12 cell line (Figure 4d). To identify the underlying mechanism, we performed a microarray screen to quantify cell death/proliferation pathway genes in antagomir‐425 transfected PC12 cells. We found that PI3K‐Akt signaling was significantly suppressed by miR‐425 inhibition (Figure 4e). Further, with Western blot, we found that consistent with microarray pathway assay, Antagomir425 inhibited PI3K‐Akt signaling and its effects could be reversed by PI3K‐Akt pathway activator SC79. Meanwhile, Agomir425 promoted Akt phosphorylation and its effects could be inhibited by PI3K‐Akt pathway inhibitor LY294002 (Figure S5). The results confirmed a causal relationship between miR‐425 and PI3K‐Akt pathway. Moreover, we found that PTEN, a suppressor of PI3K phosphorylation, was a putative target of miR‐425 mediating the inactivation of PI3K‐Akt signaling (Figure 4f). In line with the post‐transcriptional mechanism of miRNA, miR‐425 seed targeted PTEN 3′ UTR as revealed by target prediction (Figure 4g). A luciferase activity assay confirmed that antagomiR‐425‐mediated miR‐425 inhibition induced luciferase activity of a construct with PTEN 3′UTR element, whereas PTEN 3′UTR mutation decreased luciferase activity (Figure 4g).

In miR‐425 loss‐ and gain‐of‐function experiments, miR‐425 knockdown increased PTEN expression and decreased Akt phosphorylation. In contrast, miR‐425 overexpression using agomiR‐425 transfection decreased PTEN expression and increased p‐Akt level (Figure 4h), whereas, miR‐425 deficiency increased the expression of PTEN in the brain of Mir425^+/−^ mice as well (Figure 4i). Taken together, these results demonstrate that miR‐425 deficiency mediates PI3K‐Akt signaling inactivation, decreased neurogenesis, and cell proliferation via directly targeting PTEN.

### 2.5 miR‐425 deficiency results in impaired synaptic plasticity and cognitive impairment in mice

**FIGURE 5:**
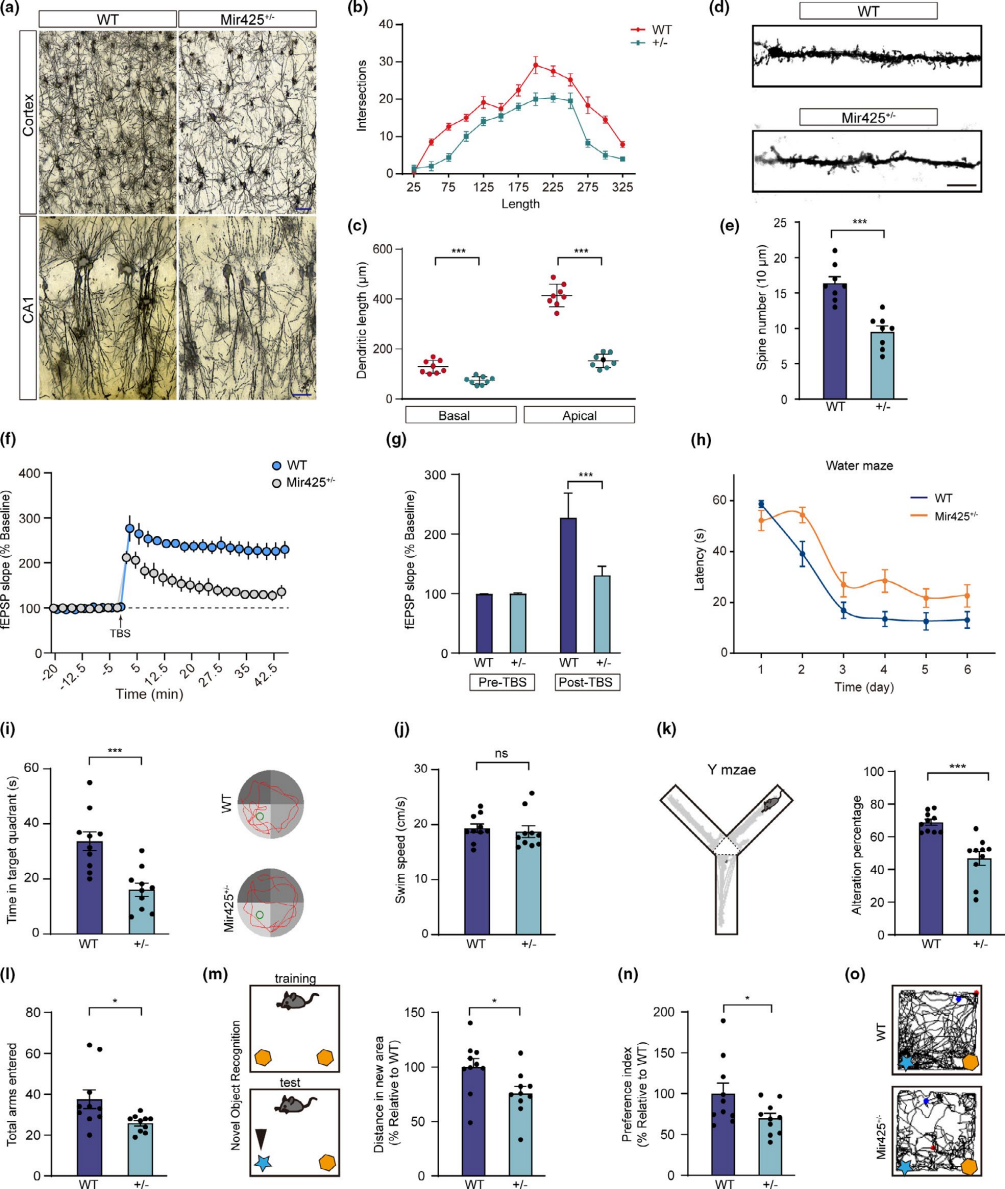
miR‐425 deficiency results in impaired synaptic plasticity and cognitive impairment in mice. (a) Golgi staining reveals dendritic growth of 15‐month‐old Mir425^+/−^ and WT mice, *n* = 6–8 per group. Scale bars, 50 μm. (b) Sholl analysis of dendritic complexity in Mir425^+/−^ and WT mice. (c) Analysis of basal and apical dendritic length in Mir‐425^+/−^ and WT mice. Scale bars, 20 μm. (d, e) Spine density of Mir425^+/−^ and WT mice, *n* = 8 per group. (f) Field excitatory postsynaptic potential (fEPSP) traces before and after LTP detection. (g) Average LTP amplitude pre‐ and post‐TBS in Mir425^+/−^ and WT mice. (h, i) Morris water maze showing escape latencies, time in target quadrant and swimming path of 15‐month‐old Mir425^+/−^ and WT mice, *n* = 10 per group. (j) Swimming speed of mice in Morris water maze. (k, l) Y‐maze tests showing the number of arm entered and Y‐maze alternation of 15‐month‐old Mir425^+/−^ and WT mice, *n* = 10 per group. (m–o) NOR tests showing the total distance of novel object exploration and discrimination ratios in 15‐month‐old Mir425^+/−^ and WT mice, *n* = 10 per group. Data were presented as mean ± SEM. Two‐tailed unpaired Student's *t* test. ***p < 0.001; **p < 0.01; *p < 0.05

Previous studies have reported that PTEN antagonizing the activity of PI3K‐Akt signaling mediates Aβ‐induced neuronal growth impairment and synaptic dysfunction in AD (Jurado et al., 2010; Knafo et al., 2016). To further elucidate the potential role of miR‐425‐mediated PTEN regulation in synaptic plasticity, we investigated dendritic spine structure in vivo using Golgi staining in Mir425^+/−^ mouse brains. We found that the spine densities of basal and apical spines were significantly decreased in miR‐425^+/−^ mice, suggesting that miR‐425 deficiency induced neurite outgrowth deficits (Figure 5a–c). Moreover, compared to WT mice, the neurons of Mir425^+/−^ mice exhibited decreased dendritic spine lengths and complexity. Consistently, dendritic spine densities were also decreased in Mir425^+/−^ mice (Figure 5d,e). IHC staining demonstrated that the synapse‐associated protein PSD95 and synaptophysin were decreased in Mir425^+/−^ mice (Figure S6A‐C). To further confirm the effect of miR‐425 loss on neurite outgrowth, we transfected primary neurons with miR‐425 shRNA. Results demonstrate that miR‐425 deficiency leads to impaired neurite outgrowth and decreased synapse‐associated protein PSD95 (Figure S6D–H).

Given findings of impaired synaptic development, we next examined the effects of miR‐425 deficiency on neuronal activity and plasticity. Electrophysiology revealed that long‐term potentiation (LTP) in hippocampus slices was significantly attenuated in Mir425^+/−^ mice compared to WT mice (Figure 5f). Moreover, the amplitude of LTP after TBS induction was significantly decreased (Figure 5g). These results suggest that miR‐425 deficiency impairs synaptic plasticity and miR‐425 may play a role in regulation of learning and memory.

Therefore, we explored whether miR‐425 deficiency could lead to behavioral impairments. In the Morris water maze, the progress of Mir425^+/−^ mice was much slower during acquisition and the latency was increased relative to WT (Figure 5h). The time mice spent in the target quadrant was also decreased in miR‐425‐deficient mice compared to WT (Figure 5i, Figure S6I). Importantly, swimming speed was similar between WT and Mir425^+/−^ mice (Figure 5j). These findings suggest that learning and memory are indeed impaired in miR‐425‐deficient mice. To further validate this observation, we assessed mouse performance in Y‐maze exploration, and both the number of arm entered and alteration percentage were decreased in Mir425^+/−^ mice versus WT (Figure 5k,l, Figure S6J). Similarly, the results of NOR test showed that Mir425^+/−^ mice have lower discrimination ratios for the total time of novel object exploration (Figure 5m–o). These findings further indicate that miR‐425 deficiency impairs memory dependent on the hippocampus.

### 2.6 miR‐425 deficiency changes global RNA expression profiles associated with AD pathological alterations

**FIGURE 6:**
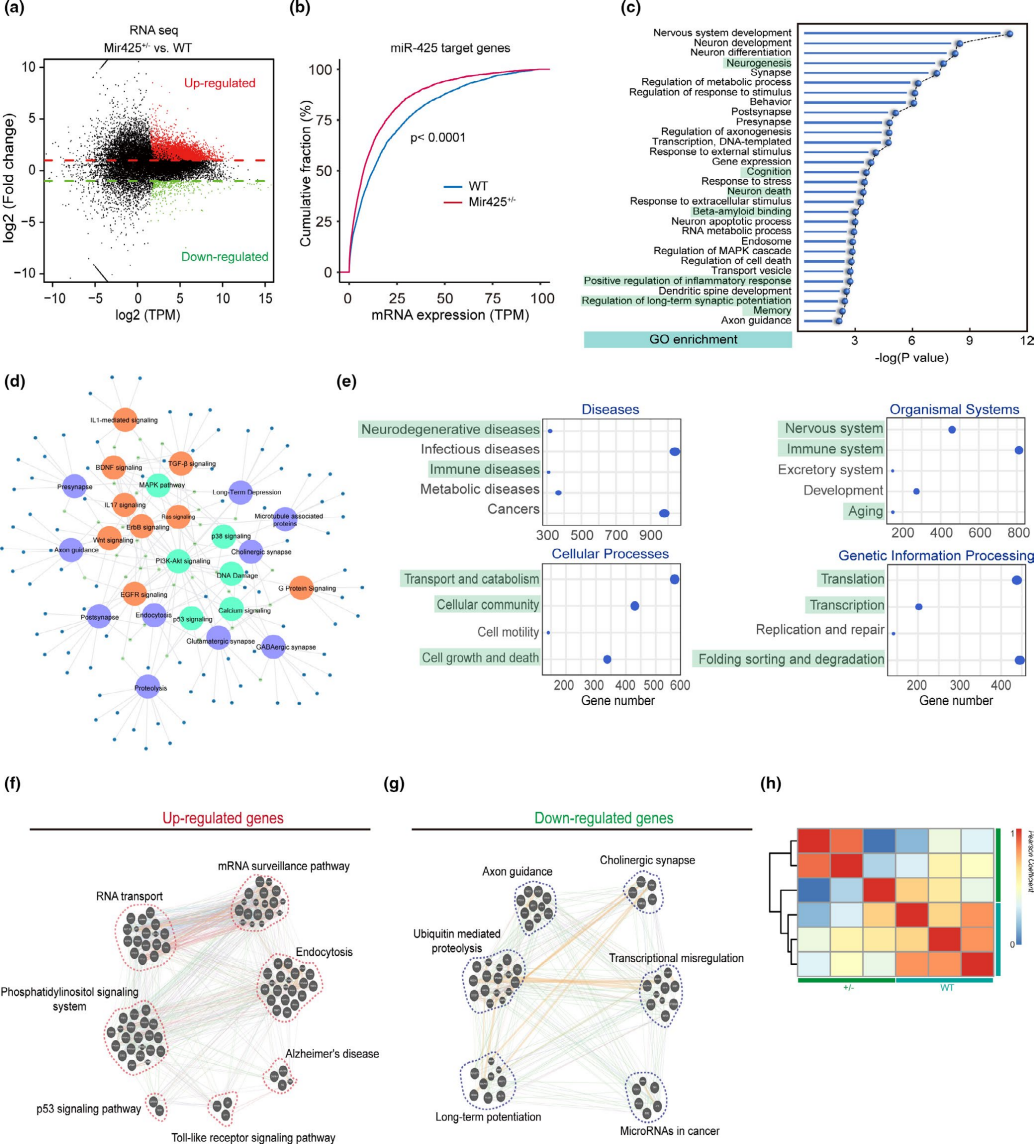
miR‐425 deficiency changes global RNA expression profiles associated with AD pathological alterations. (a) Scatter plot of RNA‐seq on the hippocampal RNA of Mir425^+/−^ and WT mice, *n* = 3 per group. (b) Cumulative distributions of target mRNAs expressions of miR‐425 in 15‐month‐old Mir425^+/−^ and WT mice. *p* Values were calculated by two‐sided Kolmogorov–Smirnov (K‐S) test. (c, d) GO enrichment analysis and gene network of DEGs. (e) GO enrichment analysis of DEGs on human diseases, organismal systems, cellular processes, and genetic information processing. (f) Enriched pathways of up‐regulated genes. (g) Enriched pathways of down‐regulated genes. (h) Pearson's correlation analysis of the transcripts in the hippocampus of 15‐month‐old Mir425^+/−^ and WT mice

To characterize miR‐425 deficiency‐mediated molecular changes at transcriptional level, we compared the mRNA profiles of WT and miR‐425‐deficient mice using unbiased RNA‐seq. We identified 1250 up‐regulated genes and 763 down‐regulated genes, and confirmed that miR‐425 deficiency led to derepression of target mRNAs (Figure 6a,b), suggesting that miR‐425 deficiency results in a gain of new functions, which may exert potent biological effects associated with neurodegeneration. To this end, KEGG analysis of DEGs was performed, cross‐referencing different gene expression lists with the GO database (Figure 6c,d). To be more precise, for human diseases, this analysis suggests that the DEGs are indeed involved in neurodegenerative diseases, immune diseases, and cancers. Consistently, these DEGs may modulate the function of the nervous, immune, and excretory systems, and also the processes of development and aging. Furthermore, in Mir425^+/−^ mice, a variety of cellular processes such as cellular transport, metabolism, cell growth, and death may be dysregulated. Specifically, miR‐425 deficiency led to impaired genetic information processing including translation, transcription, DNA regulation, and DNA repair. Collectively, KEGG analysis of DEGs revealed an association of miR‐425 deficiency with cellular process dysregulation and neurodegeneration (Figure 6e). To further provide mechanistic insight, we employed gene set enrichment and showed that up‐regulated genes were associated with RNA transport, RNA surveillance, phosphatidylinositol signaling, and AD (Figure 6f). In contrast, down‐regulated mRNAs were most significantly enriched in proteolysis, LTP, axon guidance, cholinergic synapses, transcriptional dysregulation, and miRNAs associated with cancer (Figure 6g). In addition, hierarchical clustering analysis showed significant gene alteration between miR‐425‐deficient and WT mice (Figure 6h). In conclusion, transcriptomic analysis of miR‐425‐deficient mice confirms that many dysregulated genes downstream of miR‐425 are linked to AD pathogenesis through their roles in proteolysis, endocytosis, LTP, cholinergic impairment, transcriptional dysregulation, and promotion of cell death. The data also confirm the specific regulatory role of miR‐425 in AD pathogenesis.

### 2.7 AgomiR‐425 oligonucleotide treatment ameliorates pathological and behavioral phenotypes in the APP/PS1 AD model

**FIGURE 7:**
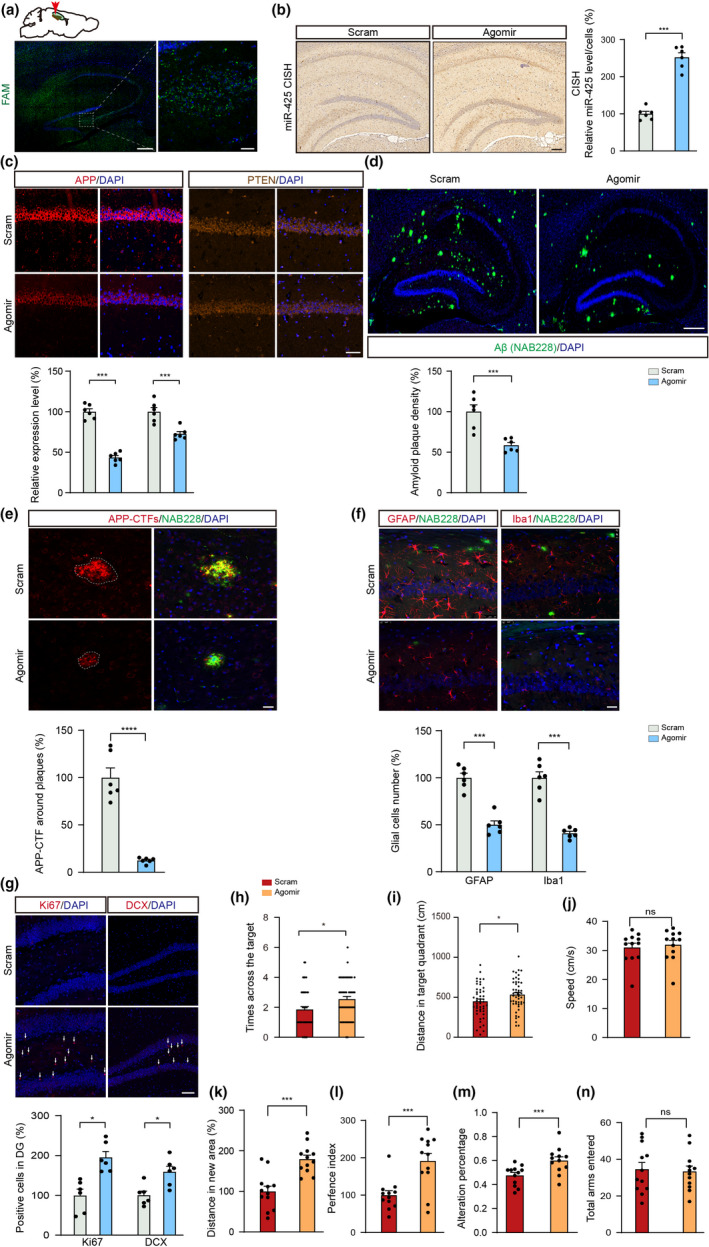
AgomiR‐425 oligonucleotide treatment ameliorates pathological and behavioral phenotypes in APP/PS1 mice. (a) Stereotactic injection of FAM‐labeled oligonucleotide in the hippocampus of 6‐month‐old APP/PS1 mice. Scale bars, 200 μm (left), 20 μm (right). (b) Chromogenic in situ hybridization and quantification of miR‐425 in hippocampus of APP/PS1 mice. Scale bars, 100 μm, *n* = 6 per group. (c) Immunofluorescence and quantification of APP and PTEN in AgomiR‐425‐ and scramble‐treated APP/PS1 mice, *n* = 6 per group. Scale bars, 50 μm. (d) Immunofluorescence and quantification of amyloid plaques in AgomiR‐425‐ and scramble‐treated APP/PS1 mice, *n *= 6 per group. Scale bars, 100 μm. (e, f) Immunofluorescence and quantification of APP‐CTFs, GFAP and Iba1 in AgomiR‐425‐ and scramble‐treated APP/PS1 mice. Scale bars, 25 μm. (g) Immunofluorescence and quantification of Ki67‐ and DCX‐positive neurons in AgomiR‐425‐and scramble‐treated APP/PS1 mice. Scale bars, 50 μm. (h–j) MWM data showing times across the target, distance in target quadrant for four times and swimming speed of APP/PS1 mice, *n* = 12 per group. (k, l) NOR tests showing the total distance of novel object exploration and preference index of APP/PS1 mice, *n* = 12 per group. (m, n) Y‐maze tests showing alternation percentage and the number of arm entered of APP/PS1 mice, *n* = 12 per group. Data were presented as mean ± SEM. Two‐tailed unpaired Student's *t* test. ***p < 0.001; *p < 0.05

As miR‐425 was decreased in human AD brains and in the APP/PS1 mouse model, AgomiR‐425 oligonucleotide was designed and synthetized, and then evaluated for the potential use in treating AD. AgomiR‐425 oligonucleotide was injected into the hippocampus of 6‐month‐old APP/PS1 mice (Figure 7a). Two months after the injection, the level of miR‐425 in hippocampus was increased by twofold to threefold (Figure 7b). AgomiR‐425 treatment led to significant reductions in target gene expression of APP and PTEN (Figure 7c), and the density of amyloid plaques (Figure 7d) and the volume of APP‐CTFs around amyloid plaques (Figure 7e) in the hippocampus of APP/PS1 mice, indicating that miR‐425 supplementation reduces Aβ production and aggregation by attenuating BACE1‐mediated APP cleavage. In addition, reactive microglia and astrocytes (Figure 7f) and neuritic dystrophy around amyloid plaques co‐labeled by NAB228 and LAMP1 (Figure S7A,B) were significantly reduced by AgomiR‐425 treatment, suggesting that the neuroimmunopathological response and neuron damage are attenuated by the transfer of miR‐425. Furthermore, AgomiR‐425 supplementation increased neurogenesis as measured by the number of Ki67‐positive, and DCX‐positive cells (Figure 7g), and up‐regulated synapse‐associated proteins (Figure S7C,D) and spine densities in the hippocampus of APP/PS1 mice (Figure S7E,F). Collectively, these data suggest that AgomiR‐425 oligonucleotide treatment ameliorates AD‐associated complex pathological changes in the APP/PS1 mouse model.

After confirming the improvement of AD‐like pathology of AgomiR‐425‐treated APP/PS1 mice, we next evaluated the effects of AgomiR‐425 oligonucleotide on spatial memory in these AD mice. First, we confirmed that AgomiR‐425 oligonucleotide treatment has no significant influence on wildtype mice on learning and memory (Figure S8). However, in the treatment of APP/PS1 mice, the AgomiR‐425 group rather than the control group showed higher crossing times and swam more distance in the target quadrant, indicating better memory in probe trials of the MWM (Figure 7h–j). Similarly, performance in the NOR test revealed that increased miR‐425 overexpression corresponded to a higher exploration discrimination ratio in APP/PS1 mice (Figure 7k,l). The Y‐maze test also demonstrated that AgomiR‐425 oligonucleotide treatment increased alternation ratios relative to the control, but the total number of arms entered was similar (Figure 7m,n). Collectively, AgomiR‐425 supplementation shows protective effects on the spatial learning deficits in APP/PS1 mice.

## 3 DISCUSSION

### 3.1 The spatially defined transcriptome regulates neurodegenerative pathologies induced by amyloid plaques

Given the important role of amyloid plaques in AD pathogenesis, research efforts have focused on identifying how amyloid plaques act in the pathogenic mechanism(s) initiating cerebral neurodegeneration. According to the amyloid cascade hypothesis, promoting the clearance of amyloid or reducing its aggregation has been the goals of preclinical and clinical studies for several decades (Mullard, 2016). Recently, numerous studies found that amyloid pathology can spread in the AD brain like prions (Honda, 2018; McAllister et al., 2020). This is not inconsistent with senile plaques influencing the brain microenvironment and facilitating more amyloid‐β production, aggregation and spreading. However, little was known about how amyloid plaques affect the brain microenvironment in vivo even though it is well‐established that polymeric Aβ can reduce cell viability and trigger cytokine release in in vitro experiments (Salminen et al., 2008). Our study provides new insights into the relationship between amyloid plaque formation and the development of AD pathologies. Amyloid plaque development is the one of earliest pathologies that forms in the AD brain, and we find cellular and molecular evidence for miR‐425‐dependent feed‐forward changes in the APAM, which promote further pro‐amyloidogenic immunologic, neuronal, and glial cellular phenotypes in mice. This is a smoking gun for miR‐425 central roles in the transition of AD from a biochemical to a cellular phase, as described by De Strooper and Karran (De Strooper & Karran, 2016). APAM downstream of miR‐425 deficiency, just like cancer‐associated TME and TIME (Fane & Weeraratna, 2020), is the means by which the brain microenvironment becomes increasingly heterogeneous, due to both aging and initial plaque deposit‐related signaling. This signaling promotes cellular phenotype changes that further lock the brain into the disease course of AD. The transcriptome of APAM from plaques supports the hypothesis that early formation of pioneer amyloid plaques leads to focal brain heterogeneity, promoting APAM. The APAM consists of multiple specific neurodegenerative signature pathologies associated with senile plaques that contribute to molecular commitment of the amyloid‐accumulating brain toward the overt neurodegenerative phenotype of AD via a cascade of pathological cellular‐specific changes. Our experiments allow the conclusion that these changes are likely subtle at tissue level, but become obvious when neurons are isolated from different microenvironments and compared. In APAM, the spatial transcriptome becomes dysregulated due to miR‐425 loss, and these changes result in a cascade of pathological changes associated with the cellular phase of AD.

In this study, we identified that many AD‐associated pathologies including cell death, inflammatory responses, and endolysosomal dysfunction were spatially colocalized with amyloid plaques. Isolation of APAM followed by RNA sequencing and integrative omic analysis confirms that early amyloid plaque aggregation triggers localized APAM‐specific transcriptomic changes and mediates feed‐forward to accelerated APP amyloidogenic processing and other later‐appearing neurodegenerative pathologies including neuroinflammation, reactive astrocytosis, microglial phenotype switching, and neuronal death. These results highlight the complexity of APAM and reveal a lynchpin of how amyloid aggregation in the brain can lead to neurodegeneration with overt brain dysfunction and cognitive impairment. Our work further highlights a point in the amyloid cascade which is amenable to therapy that has the potential to defuse the cascade before the commitment to an overt AD phenotype and symptoms, which in humans only happens years after initial amyloid deposition.

Specifically, we identify loss of miR‐425 as an event during AD progression that is a key driver of overt neurodegeneration. miR‐425 is thus implicated in the suppression of spatial transcriptomic network changes and neurodegeneration and has the role of a suppressor, rather than driver, via its enforcement of a normal spatial transcriptome in brain areas, and neurons in particular that are otherwise susceptible to the APAM. Through prediction of a target gene network, we find significant enrichment of biological processes and signaling pathways associated with AD among gene products potentially regulated by miR‐425. The level of miR‐425 was significantly decreased in the brain of symptomatic AD patients and in the APP/PS1 aged mouse model, due to higher methylation on the promoter of the miR‐425 gene.

### 3.2 The role of miR‐425 loss in neurodegeneration

Further illuminating the role of miR‐425 in AD pathogenesis, we adopted CRISPR/Cas9 gene editing to generate miR‐425^+/−^ mice to mimic the miR‐425 deficiency in overt AD. Several deregulated miRNAs have been documented in different lines of evidence to be associated with Aβ production (Hebert et al., 2008). But in our current and prior works, for the first time, we identify miR‐425 targets including both APP and BACE1, where miR‐425 loss enhances both amyloidogenic processing and Aβ production. Moreover, we also found that elevated APAM‐associated activation of astrocytes and microglia occurs as part of the phenotypes of these miR‐425‐deficient mice. Even though glial cell phenotype switching during AD pathogenesis remains controversial if not incompletely defined, multiple lines of evidence suggest that they play a neurotoxic role by secreting cytokines and reactive oxygen species (Mhatre et al., 2015).

Recent studies revealed that newly formed neurons in the hippocampus are persistent but decreased with aging, suggesting that impaired neurogenesis may lead to memory deficits in AD (Moreno‐Jimenez et al., 2019), and miR‐425, among a cluster of five miRNAs, became elevated in plasma in healthy centenarians compared to younger individuals, suggesting the role of miR‐425 in healthy aging (Olivieri et al., 2012). Our observations of impaired NPC proliferation in miR‐425‐deficient mice provide two possible insights into the role of miR‐425 in AD‐related pathogenesis. First, previous findings revealed that APP and its cleaved fragments impair neurogenesis (Ma et al., 2008), and this is consistent with our finding APP expression and amyloidogenic cleavage are increased in the brain of miR‐425‐deficient mice. Second, our investigations further confirmed that the PI3K‐Akt signaling pathway is implicated in miR‐425‐induced cell proliferation and that miR‐425 directly targets the 3′ UTR of PTEN, regulates PTEN levels, and mediates the inhibition of PI3K‐Akt signaling. Other studies also reported PTEN up‐regulation and PI3K‐Akt signaling dysfunction in neurodegenerative diseases, including AD (Jurado et al., 2010; Kitagishi et al., 2014). Our current data confirm the proliferative effects of normal miR‐425 levels in the brain and demonstrate that miR‐425 loss is responsible for PI3K‐Akt signaling suppression and impaired NPC proliferation. Moreover, we confirm that miR‐425 loss induces dendritic spine defects and impaired synaptic plasticity. Finally, we find that aged miR‐425‐deficient mice have overtly impaired learning and memory. Taken together, miR‐425 loss following initial plaque deposition is the opening of the floodgates that mediates the ability of the APAM to cascade into a cellular phase of AD (Figure S9). Importantly, in our transgenic mouse model of miR‐425 deficiency, we replicate APAM changes and neurodegenerative pathologies without amyloid plaque formation. This highlights the potential that miR‐425‐mediated APAM changes, rather than amyloid plaque itself, are etiologically necessary for overt neurodegeneration and symptoms of AD, though further work will be required.

### 3.3 Modifying APAM changes ameliorate neurodegenerative pathologies and rescues memory deficits

Pharmacologic miR‐425 replacement targeting the normalization of APAM gene networks is possible, as we find that miR‐425 supplementation reverses the changes of target genes associated with disease and attenuates amyloid plaque formation in APP/PS1 mice. AgomiR‐425 improved the learning and memory of these AD model mice, and rescued synaptic abnormalities and memory deficits. These data suggest that miR‐425 replacement has neuroprotective proprieties and attenuates conditions that allow for cellular cascades that engage a commitment of the brain to develop AD‐associated pathologies. Therefore, our findings reveal that developing therapies that reverse APAM changes, and APAM‐initiated cascades, may be an opportunity to prevent the conversion from asymptomatic AD to overtly symptomatic AD. The highly conserved regulator miR‐425 appears to be one suitable target in this regard. Insights into the roles of miRNAs in other diseases, particularly in cancer, miRNAs acting as tumor suppressors or oncogenes (oncomiRs), and miRNA mimics and molecules targeted at miRNAs (antimiRs) have shown promise in preclinical experiments (Bandiera et al., 2015). Several miRNA‐targeted therapeutics have reached clinical phase I‐II trials for treating cancer and hepatitis (Miya Shaik et al., 2018; Rupaimoole & Slack, 2017). We look forward to future clinical trials of miRNAs, including miR‐425 for the prevention of AD conversion from marker‐positive asymptomatic individuals.

## 4 CONCLUSION

In summary, this study elucidates that a loss of miR‐425 is required for APAM to affect cellular cascades across cell types of AD brain, namely the hallmark molecular, cellular, and behavioral abnormalities associated with AD‐like neurodegeneration, and those changes are sufficient to impede learning and memory capacity in this model. Moreover, our findings suggest that modifying APAM changes and responsiveness of neurons via miR‐425 replacement could be an attractive therapeutic strategy for treating neurodegenerative diseases, including AD.

## 5 MATERIALS AND METHODS

### 5.1 Mice

APP^swe^/PSEN1ΔE9 (APP/PS1), Mir425^+/−^ mice and littermate control were obtained from the Model Animal Research Center of Nanjing University. All the mice in the study were male. APP/PS1 mouse overexpresses human APP Swedish mutation (K670N/M671L) and mutant presenilin‐1 with exon 9 missing. Heterozygous Mir425^+/−^ mice (maintained on C57BL/6 background) were generated with the CRISPR/Cas9 technique. Briefly, guide RNAs were designed targeting the 5′ and 3′ ends of mouse Mir425. A mixture of gRNA and Cas9 mRNA was microinjected into embryos of C57BL/6 mice at one‐cell stage. The injected embryos were implanted into C57BL/6 mice to generate F0 mice. Then, F0 mice were randomly selected to cross with C57BL/ 6 mice to generate F1 mice. The genotype was confirmed using PCR. The primers used for Mir425^+/−^ mice genotyping are as follows: the forward primer: 5′‐ATGGTGGCAGTCAGAGGCGA‐3′; the reverse primer 5′‐GTGATGATGAGAAGACCCAA‐3′. All animal used in this study were male with age‐matched wildtype controls, and the experiment protocols were approved by the Ethics Committee of Shanghai Jiao Tong University School of Medicine.

### 5.2 Primary neuronal culture

For primary cortical neuronal cultures, pups were decapitated individually and hippocampi were gently triturated in Hanks’ medium with Pasteur pipettes. After trypsinization and dissociation, cells were suspended and plated on 6‐well plates (6 × 10^5^ cells/well) pre‐coated with poly‐d‐lysine. The cells were cultured in Neurobasal media (Invitrogen) supplemented with B27 and l‐glutamine in a humidified incubator with 5% CO_2_ at 37°C. Half of the culture medium was replaced every 3 days.

### 5.3 Cell lines

PC12 cell line was obtained from the Cell Line Resource Center of the Chinese Academy of Sciences (Shanghai, China). PC12 cells stably transfected with APP695 Swedish mutation were cultured in Dulbecco's modified Eagle's medium (DMEM, ThermoFisher) with 10% FBS (ThermoFisher) and 1×penicillin‐streptomycin. Cell cultures were maintained in a humidified atmosphere of 5% CO_2_ at 37°C. Cell viability was measured using Cell Titer Luminescent Cell Viability Assay (Promega, USA) in 96‐well plates in accordance with manufacturer instructions.

### 5.4 Human brain samples

Postmortem brain tissues were obtained from the human brain bank of Peking Union Medical College (PUMC). Samples include 6 controls and 6 AD patients with clinical diagnosis and neuropathological confirmation before examinations. Detailed information for human brain samples including age, sex, diagnosis, and pathological stages can be found in Table S1. This study had ethical approval from ethical committees of PUMC and Shanghai Jiao Tong University School of Medicine.

### 5.5 Immunohistochemistry

For fluorescence immunohistochemistry, mouse brains were mounted and sliced into 30 μm sections. Sections were subsequently washed for 10 min with PBST plus 0.1% Triton X‐100, blocked for 1 h in 10% normal goat serum, and incubated overnight at 4°C with the primary antibodies. Primary antibodies used in this study: APP (22C11, ThermoFisher, 14‐9749‐82), APP (Y188, Abcam, ab256586), APP C‐terminus (A8717, Sigma), β‐Amyloid (NAB228, Cell Signaling Technology, 2450), BACE1 (Abcam, ab108394), p16 (Abcam, ab51243), p21 (Abcam, ab109520), Caspase‐3 (Abcam, ab13847), Cleaved Caspase‐3 (Abcam, ab2302), LAMP1 (Abcam, ab24170), EEA1 (Abcam, ab2900), Rab5 (Abcam, ab18211), NeuN (Abcam, ab177487), GFAP (Abcam, ab7260), Iba1 (Abcam, ab178846), PTEN (Abcam, ab32199), PSD95 (Abcam, ab18258), Doublecortin (DCX) (Santa Cruz, sc‐8066), Ki67 (Abcam, ab15580), Nestin (Biolegend, 656802), Akt (pan) (Abcam, ab8805), AKT (phospho T308) (Abcam, ab38449), Synapsin I (Abcam, ab64581), Synaptophysin (Abcam, ab32127), and GAPDH (Abcam, ab8245). After washing with PBST, the sections were incubated with a mixture of secondary antibodies for detection. The following secondary antibodies were used: anti‐mouse/rabbit Alexa Fluor 488, 594, and 647 (ThermoFisher, USA). Covering with Antifade Mountant (ThermoFisher, USA), the sections were visualized. Confocal images were acquired on a Leica TCS SP8 Confocal Microscope with LAS AF software (Leica Microsystems, Germany). When images were captured on the platform, the optical and laser settings, pinhole, and resolution were empirically determined and kept constant for subsequent samples within an experiment. Images were captured with 10 ×, 20 ×, 40 ×, or 63 × objectives for all settings, and the fluorescence intensities were calculated and compared on LAS AF software. Quantification of co‐localization was analyzed with the ImageJ software.

### 5.6 Laser capture microdissection (LCM)

APP/PS1 mice were sacrificed under isoflurane anesthesia and immediately perfused with cold PBS with DEPC. Harvested brain tissues were fixed and embedded in optimal cutting temperature compound (OCT, SAKURA, Japan) and dissected into 10 μm slices and stored at −80°C until the laser capture microdissection. For amyloid plaques staining, slices were washed with 70% ethanol for 60 s, stained with 1% NAB228 and NeuN antibodies at 4°C overnight, followed by secondary antibodies incubation after washing. Then, another wash was followed for 10 min before being completely air‐dried. Around amyloids (APs) or non‐APs were microdissected with Arcturus XT Laser Capture Microdissection System from each section and collected into a single tube. Harvested cells were stored at −80°C.

### 5.7 RNA sequencing, microarray, and integrated bioinformatics analyses

For LCM samples, RNA and miRNA were extracted and purified using the LCM RNA extract kit and Dynabeads RNA purification kit (ThermoFisher, USA). Small RNA libraries were prepared using SMART Small RNA Kit (Takara, Japan) according to manufacturer's instructions. LCM samples were used to identify miRNAs signature with miRNA microarray (Agilent Mouse miRNA Microarray, Release 21.0). The RNA was purified, amplified, and labeled; the microarray hybridization was conducted according to the manufacturer's instructions. The arrays were scanned with Agilent Microarray Scanner and the raw data were normalized by an Expression Console with a fold change >2 and *p* < 0.05 were regarded as significantly different. High‐throughput sequencing was performed on an Illumina HiSeq 4000 (Aksomics, China). DESeq and EdgeR were used for differential expression between two groups. Gene ontology (GO) and KEGG analysis was performed on R software. Online GeneMANIA was used to predict the function and interaction of the differentially expressed genes (Warde‐Farley et al., 2010).

### 5.8 Droplet digital PCR (ddPCR)

The ddPCR for human brain miR‐425 quantification was performed using the ddPCR TM Supermix for Probes reagents in the QX200TM Droplet Digital TM PCR system (Bio‐Rad, CA). For quantification of miR‐425, RNA was reverse transcribed to cDNA.

The droplets were generated from QX200 Droplet Generator with a mixture of cDNA, digital PCR supermix, and TaqMan primer/probe mix. The PCR amplification was carried out with the C1000 Touch Thermal Cycler (Bio‐Rad, CA). The PCR products were quantified using QX200 droplet reader and analyzed with Quanta Soft software (Bio‐Rad, CA).

### 5.9 MicroRNA in situ hybridization

MicroRNA in situ hybridization was performed as previously described (Obernosterer et al., 2007). Mouse or human brain sections were prehybridized in hybridization buffer at 55°C for 30 min. Cy3‐ or DIG‐labeled LNA‐modified probes complementary to miR‐425 (miRCURY probe sequence: CAACGGGAGTGATCGTGTCAT, Exiqon) were added and hybridized for 16 h at 37°C. After post‐hybridization washes in hybridization buffer and 2× SSC at 37°C, the in situ hybridization signals were visualized using the fluorescence or DAB system. A scrambled control was used in experiments to confirm the specificity of the probes.

### 5.10 Quantitative reverse transcription polymerase chain reaction (RT‐PCR)

RNA was extracted and purified using standard procedures using Trizol and RNeasy mini kit. Real‐time PCR was performed with One Step RT‐PCR Kit (Takara, Japan) using Lightcycler480 (Roche, USA). Primer sequences for RT‐PCR are provided in Table S2. Quantitative mRNA or miRNA expression was compared using ΔCt (normalized to internal control) and ΔΔCt values. GAPDH mRNA and U6 levels were used as internal references.

### 5.11 Western blot

Proteins were analyzed for total protein concentration determined with BCA kit (ThermoFisher, USA). 30 μg of proteins was boiled with SDS loading buffer, loaded to a 4%–12% Tricine‐SDS‐PAGE gel, and transferred to a PVDF membrane. The membrane was blocked in 5% milk in TBST for 1 h, incubated with primary antibody in 5% BSA/TBST at 4°C overnight, and then rinsed with TBST. The membrane was incubated with goat anti‐rabbit/mouse IgG‐HRP secondary antibodies (1:1000), and images were captured and quantified by Odyssey LI‐COR imager. The blot was reprobed after stripping with a rabbit anti‐GAPDH antibody (1:5000) to control for protein loading.

### 5.12 Luciferase reporter assay

The 3′ UTRs of APP, BACE1, and PTEN were amplified and cloned into pmirGLO Dual Luciferase reporter vector (Promega, USA). These vectors were checked by capillary sequencing and transiently transfected into PC12 cells, together with control or AgomiR‐425, antagomir‐425. Cells were harvested 48 h after transfection and assayed for firefly luciferase and Renilla luciferase activities on a Synergy MX plate reader (BioTeck, USA).

### 5.13 Aβ and CTF‐β ELISA

Levels of Aβ and CTF‐β were determined from protein samples using standard procedures with human or murine ELISA plate (IBL, Japan) (Martorell et al., 2019). The signals were obtained at 450 nm using a Synergy MX plate reader (BioTeck, USA). The results were reported as Aβ or CTF‐β per milligram of total protein.

### 5.14 Mouse cytokine and cell death array assay

Brain hippocampus tissue or PC12 cells were homogenized and lysed in a buffer containing protease inhibitors. Total protein was determined using the BCA Protein Assay Reagent (ThermoFisher, USA). 1000 μg of protein and sample buffer were incubated with mouse cytokine array membrane. The signals were detected using the ECL kit according to the user manual.

### 5.15 Golgi‐cox staining

Mouse brains were fixed in PFA and then incubated in a 1:1 mixture of FD Solution A:B (FD Neuro Technologies) for 10 days in the dark. Then, brains were transferred into Solution C (2% silver nitrate solution) for 48 h incubation. Brains were dissected into 200 μm with a Leica CM1950, air‐dried for 8 h. Subsequent staining was performed according to the manufacturer's protocol. Images were captured under the microscope (Nikon, Japan). Analysis of dendrites and their lengths was performed on ImageJ/Fiji plugin.

### 5.16 Electrophysiological study

For acute brain slice preparation, mice were anesthetized and decapitated, and the brains were rapidly dissected out and placed in ice‐cold artificial cerebrospinal fluid (ACSF, composed of 119 mM NaCl, 2.3 mM KCl, 1.3 mM MgSO_4_, 26.2 mM NaHCO_3_, 1 mM NaH_2_PO_4_, 2.5 mM CaCl_2_, and 11 mM glucose; bubbled with 95% O_2_/5% CO_2_, pH 7.4). Hippocampal slices were cut into 300‐µm sections with a vibratome (Leica, Germany) and transferred to a transitional chamber containing ACSF. Hippocampal slices containing CA1 region were used. The strength of synaptic transmission was recorded to measure the field excitatory postsynaptic potentials (fEPSPs) amplitude (and the maximal slope) in the stratum radiatum of CA1 region with a glass microelectrode filled with ACSF with 3–7 MΩ resistance, which was evoked by stimulating the Schaffer collateral with a bipolar tungsten electrode. To determine the input–output relationship, a series of ascending stimuli were delivered in each hippocampus slice. The input–output relation (I‐O) was plotted. For the LTP study, only those slices with maximal evoked fEPSPs amplitude over 0.5 mV were used. fEPSPs were evoked by pulses at a 0.033 Hz stimulation frequency and with an intensity that was able to elicit a 40%–50% of the maximal fEPSP response. After at least 20 min of stable baseline recording, LTP was induced by a theta burst stimulation (TBS, 4 pulses at 100 Hz, 15 trains in 0.2 s interval) using the same stimulus intensity as for baseline recording. After LTP induction, fEPSPs were recorded for a further 45 min. The magnitudes of LTP were expressed as the mean percentage of baseline fEPSP initial slope.

### 5.17 Morris water maze

Morris water maze was performed as previously described (Hu et al., 2019). Each mouse was given 4 trials per day for 5 consecutive days with a 1‐min interval and allowed to search and find the hidden platform within 60 s. A probe test was conducted 24 h following the last training session with the platform removed from the pool. Mice performance in the pool was recorded for 60 s using a video camera for analysis.

### 5.18 Y‐maze

Spatial working memory was assessed by recording spontaneous alternation behavior in a Y‐maze with three arms (Choi et al., 2018). During the exploration in the maze, mice typically explored a new arm rather than returning to the arm that was recently entered. Each mouse was placed in the center of the Y‐maze and was allowed to move freely in three arms for 8 min. The sequence and total number of arms mice entered were recorded and later analyzed to calculate the alternation rate.

### 5.19 Novel object recognition (NOR)

NOR tests were performed in a rectangular box (60 cm × 60 cm × 45 cm). Before training, mice were handled for 2 consecutive days. Then, each mouse was placed in the empty arena for 10 min measuring the overall locomotion activity. For acquisition training, two different unfamiliar objects are placed along one side of the arena. Each mouse was allowed to explore the object for one 5 min session. 24 h after training, one of the familiar objects was replaced with a novel object. The mice were allowed to explore the objects for 5 min. The discrimination ratio was expressed as the proportion of time spent exploring the novel object compared with the time spent exploring both objects.

### 5.20 Oligonucleotide synthesis and stereotaxic injection

The local administration into mouse hippocampus using stereotaxic injection was performed as previously described (Hu et al., 2019). Oligonucleotides were designed and synthesized with the following modifications: 2 phosphorothioates on the 5′ termini, 4 phosphorothioates at the 3′ termini, 3′ termini cholesterol group and full length nucleotide 2′‐methoxy modification. Oligonucleotide sequences were as follow: AgomiR‐425: 5′‐AA UGACACGAUCACUCCCGUUGA‐3′, 3′‐AACGGGAGUGAUCGUGUCAUUUU‐5′; Scramble: 5′‐UUCUCCGAACGUGUCACGUTT‐3′, 3′‐ACGUGACACGUUCGG AGAATT‐5′.

Six‐month‐old APP/PS1 mice were anesthetized with 2% isoflurane and 2 μl of AgomiR‐425 dissolved in PBS was injected into the hippocampus using a 10 μl Hamilton syringe at a rate of 0.25 μl/min. Bilateral injection was performed stereotaxically at the following coordinates relative to bregma: AP −2.1 mm, ML ±2.0 mm, and relative to dural surface: DV −1.9 mm. The needle was held in place for 5 min following completion of injection. The mice were placed on a heating blanket until initial recovery from the anesthesia.

### 5.21 Statistical analyses

Data were expressed as mean ± SEM. To compare data between two groups, two‐tailed Student's *t* test was used. To examine significance among more than two groups, one‐ or two‐way ANOVAs were used followed by Fisher's least significant difference and Tukey's post hoc analyses when appropriate. The Kolmogorov–Smirnov (K‐S) test was used to compare the cumulative distribution. Statistical analyses were performed using R software or GraphPad Prism. Significance level was set at *p* < 0.05 for all tests.

## CONFLICT OF INTEREST

The authors declare no competing interests.

## AUTHOR CONTRIBUTIONS

YBH performed experiments, analyzed data, and wrote the manuscript. HW, Y‐FZ, and R‐JR designed the experiments, analyzed data, and wrote the manuscript. EBD helped with methodology and wrote the manuscript. XYX, SWC, QH,WYH, and RZ contributed to molecular and animal experiments. HZC and GW supervised the project, designed the experiments and revised the manuscript.

## Supporting information

Supplementary Material

## Data Availability

Authors declare that the author provides the data to requester when there is a reasonable request for all data supporting the findings.
