# Supplementary material for: microRNA‐425 loss mediates amyloid plaque microenvironment heterogeneity and promotes neurodegenerative pathologies
Source: Aging Cell. 2021 Sep 12;20(10):e13454. doi: 10.1111/acel.13454 (PMC8520725; doi:10.1111/acel.13454)

**microRNA-425 Loss Mediates Amyloid Plaque Microenvironment Heterogeneity and Promotes Neurodegenerative Pathologies**

**Supplemental Information**

**Supplementary Figures**


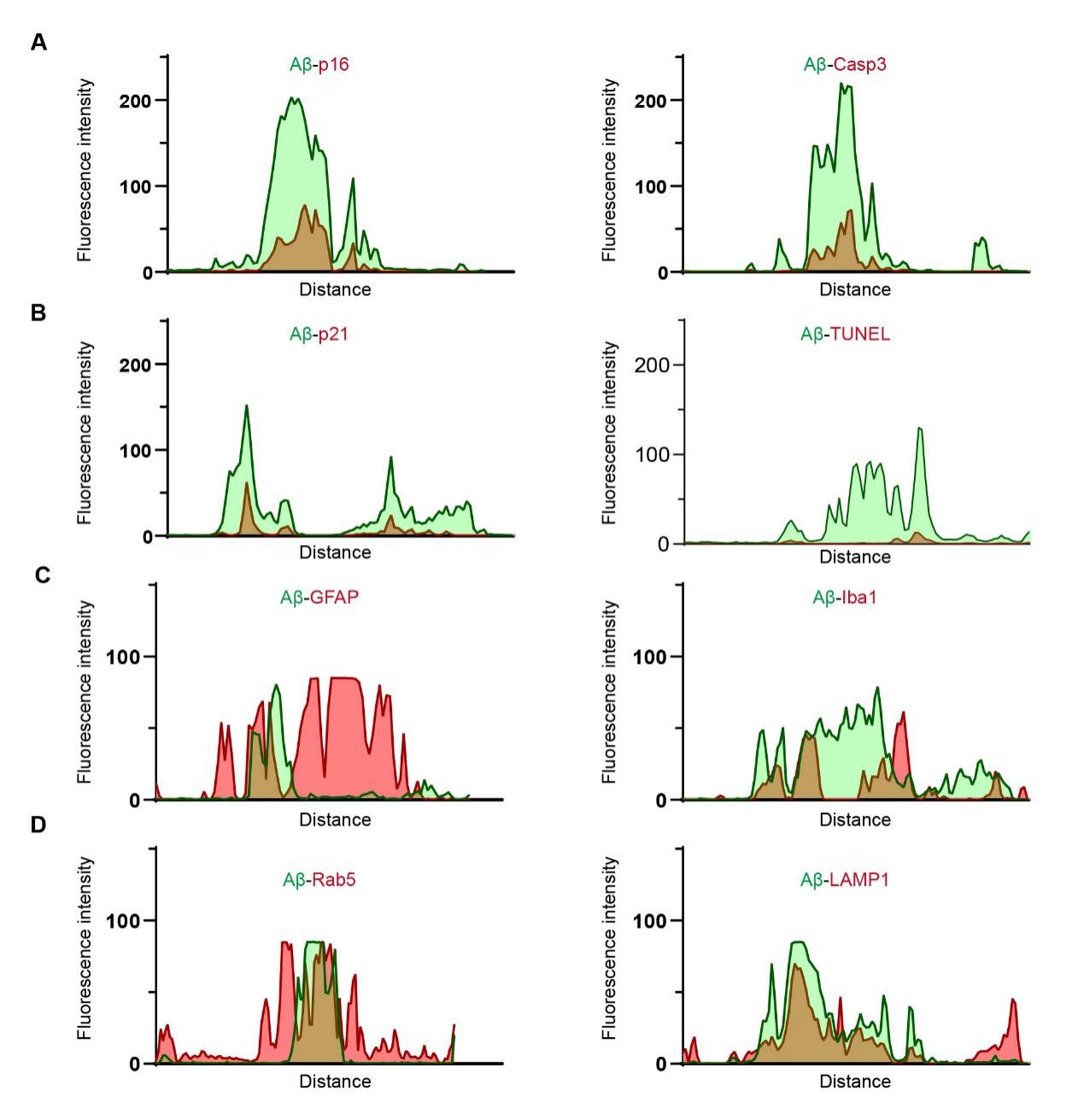


**Figure S1 Colocalization of amyloid plaques with markers of cellular senescence, neuron death, inflammation and endosomal-lysosomal dysfunction.**

Colocalization of cellular senescence (p16, p21), neuron death (Casp3, TUNEL), inflammation (GFAP, Iba1) and endosomal-lysosomal dysfunctions (Rab5, LAMP1) with amyloid plaques (Green).


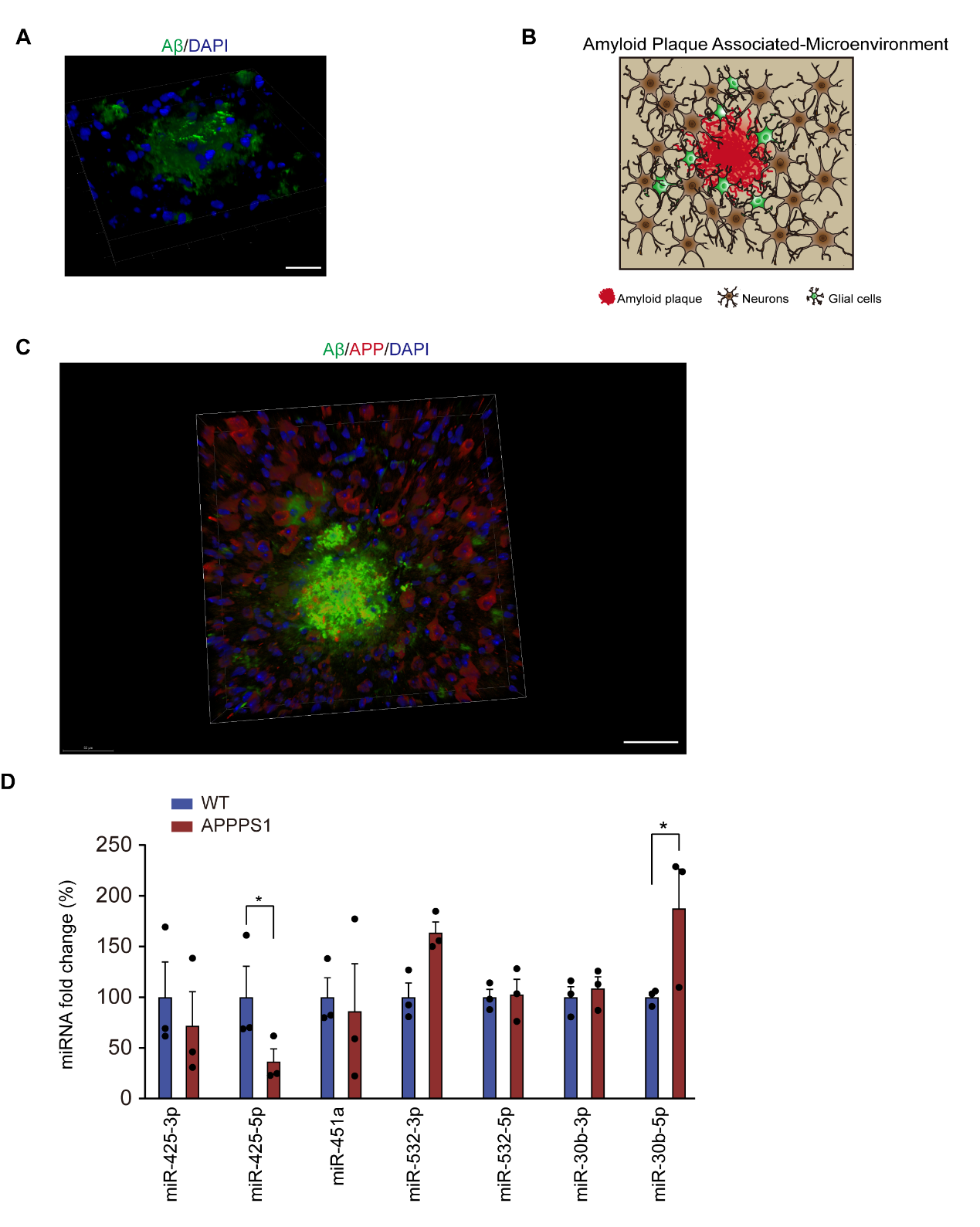


**Figure S2 Amyloid aggregation induces spatial transcriptomic changes.**

**(A)** Amyloid aggregation in the brain of APP/PS1 mice; Scale bars, 50 μm. **(B)** Schematic plot of amyloid plaques-associated microenvironment (APAM). **(C)** Colocalization of APP-positive dystrophic neurites (Red) with amyloid plaques (Green); scale bars, 50 μm. **(D)** miRNAs expression in the hippocampus of APP/PS1 and WT mice. Data were presented as Mean ± SEM. Two-tailed unpaired student’s t test. n=3 per group, *p < 0.05.


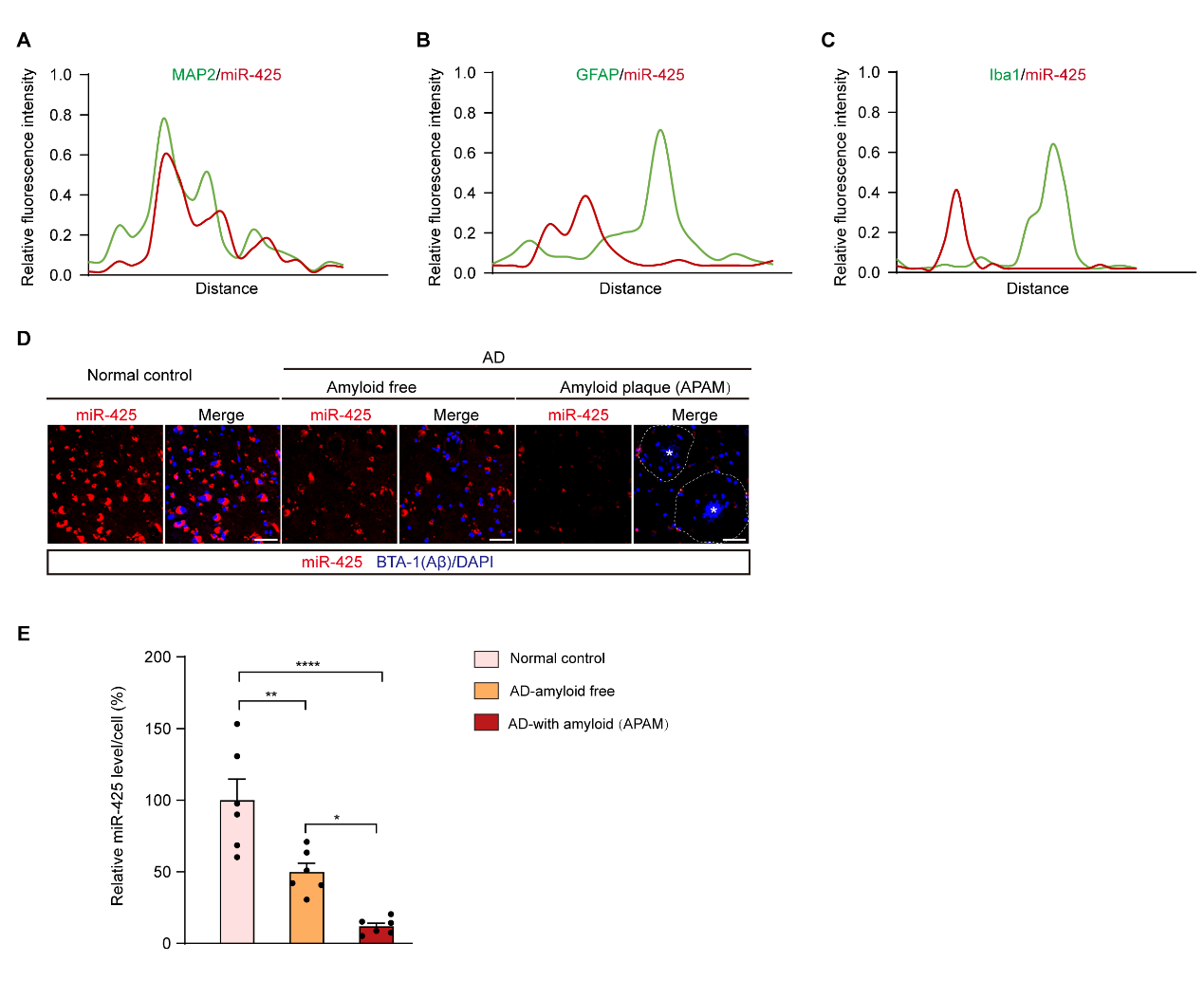


**Figure S3 miR-425 expression is negatively related to amyloid pathology.**

**(A-C)** Colocalization of neurons (MAP2, green), astrocyte (GFAP, green), and microglia (Iba1, green) with miR-425 (red). **(D-E)** *In situ* hybridization and quantification of miR-425 in the brain regions of AD patients with or without amyloid plaques aggregation. Scale bars, 20 μm.


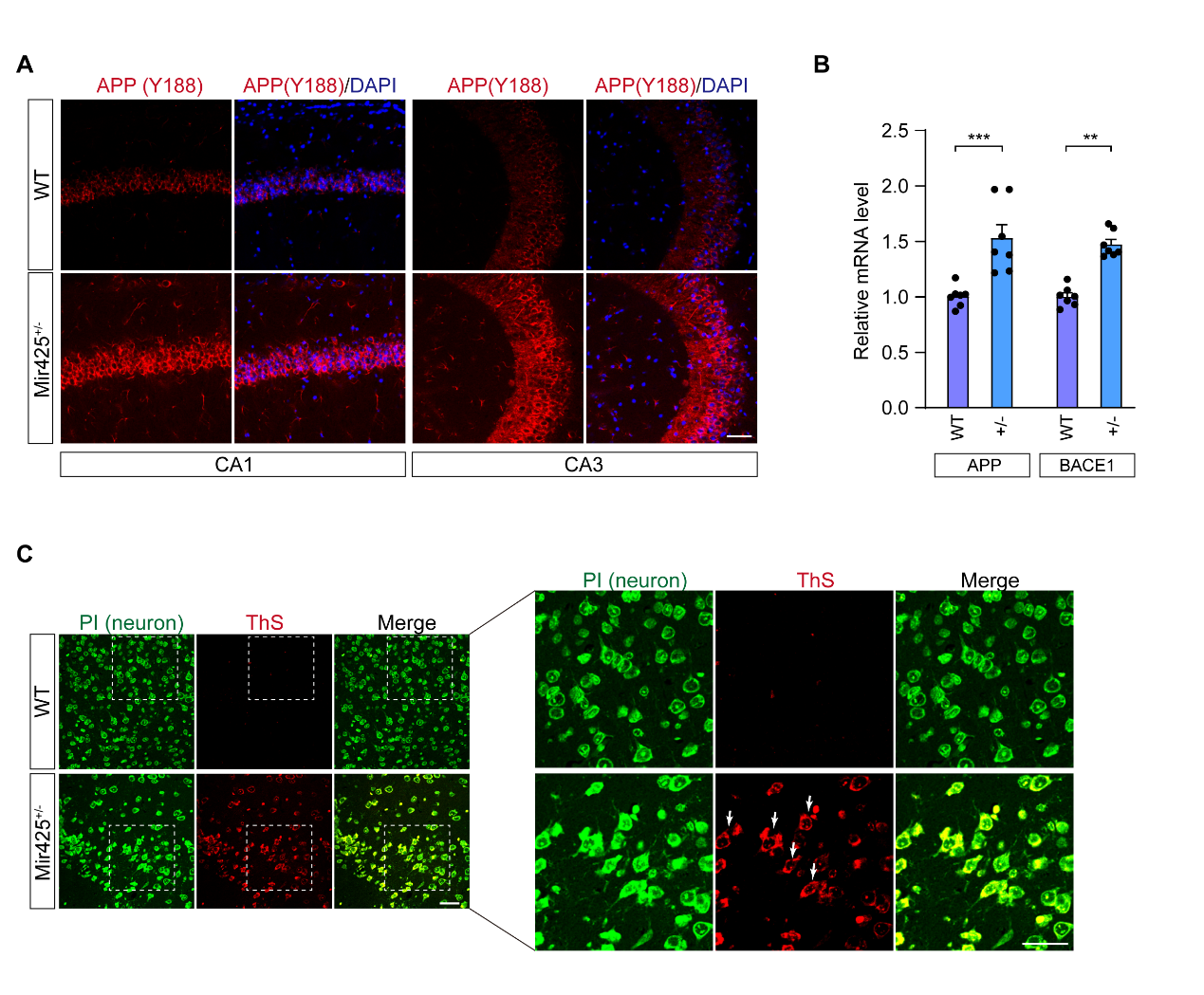


**Figure S4 miR-425 deficiency activates APP expression and Aβ production in neurons of mice.**

**(A)** Immunofluorescence of APP in the hippocampus of Mir425^+/-^ and WT mice. Scale bars, 20 μm. **(B)** RT-PCR of APP and BACE1 mRNA in the brain of Mir425^+/-^ and WT mice. **(C)** Immunofluorescence of intracellular ThS-positive Aβ in the brain of Mir425^+/-^ and WT mice. Scale bars, 20 μm. Data were presented as Mean ± SEM. Two-tailed unpaired student’s t test. **p < 0.01, ***p < 0.001.


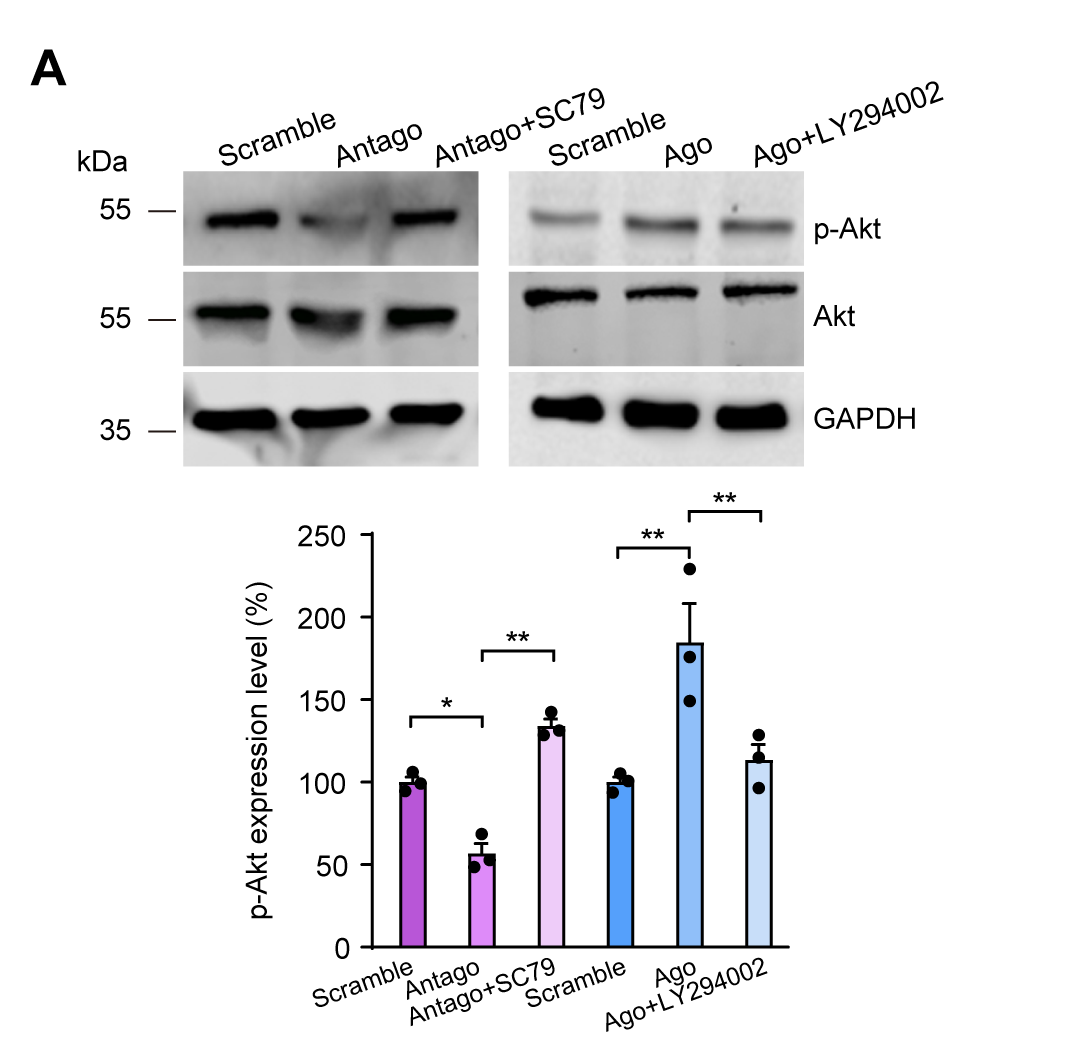


**Figure S5 miR-425 was involved in PI3K-Akt signaling regulation**

Western blot of p-Akt and Akt in PC12 cells transfected with Antagomir-425 (Anta) and Agomir-425 (Ago) with the treatment of PI3K-Akt pathway activator SC79 (10 μM) and inhibitor LY294002 (25 μM).


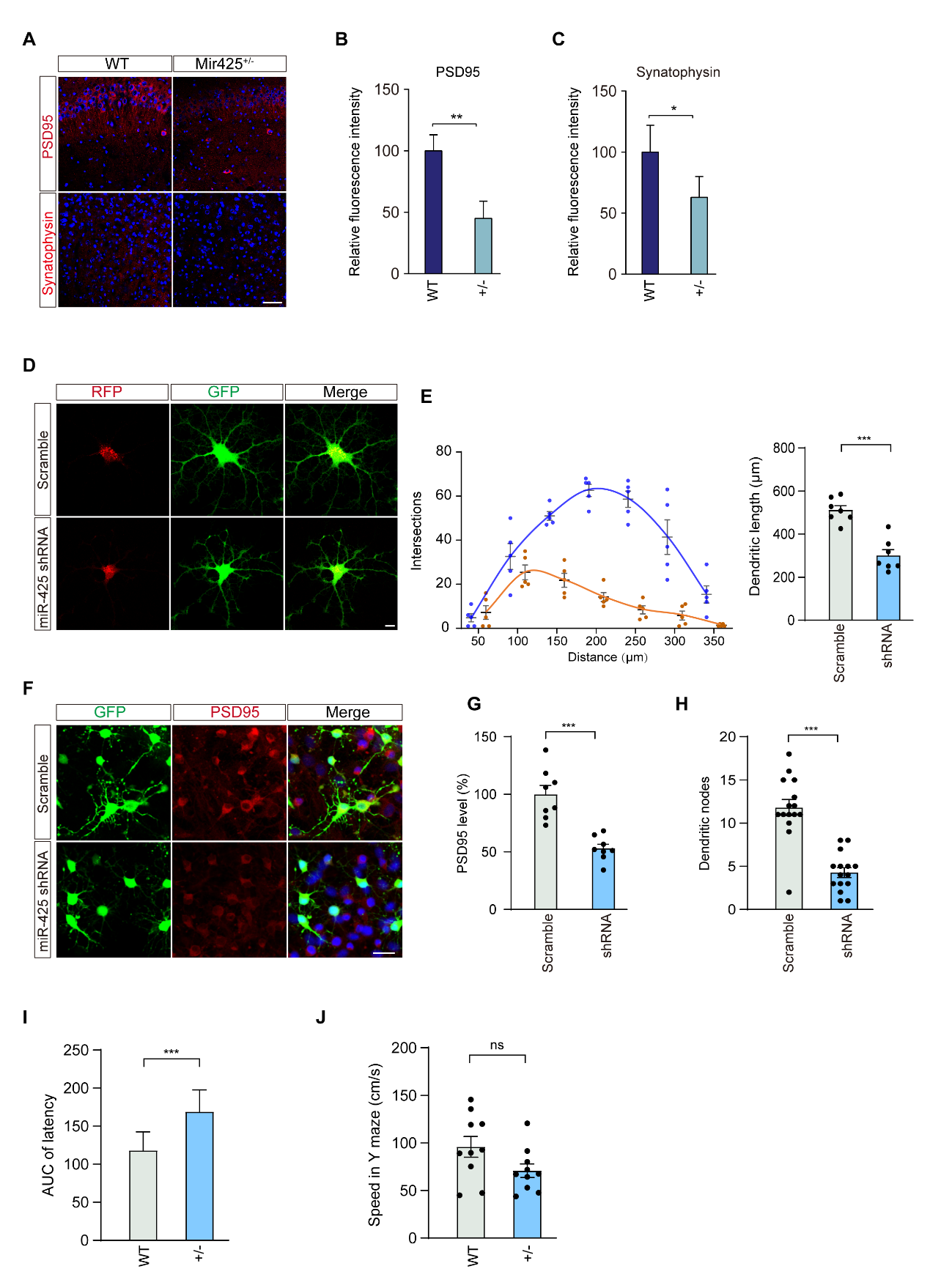


**Figure S6 miR-425 deficiency impairs synaptic plasticity.**

**(A-C)** Immunohistochemistry staining and quantification of PSD-95 and synaptophysin in Mir425^+/-^ and WT mice; Scale bars, 50 μm. **(D-E)** Impaired dendritic growth of miR-425 knockdown primary neurons; Scale bars, 10 μm. **(F-H)** Immunofluorescence and quantification of PSD95 and sholl analysis of dendritic complexity in miR-425 knockdown primary neurons. Scale bars, 50 μm. **(I)** AUC of escape latencies in Morris water maze; **(J)** Speed of mice in Y maze. Data were presented as Mean ± SEM. Two-tailed unpaired student’s t test. ***p<0.001; *p<0.05.


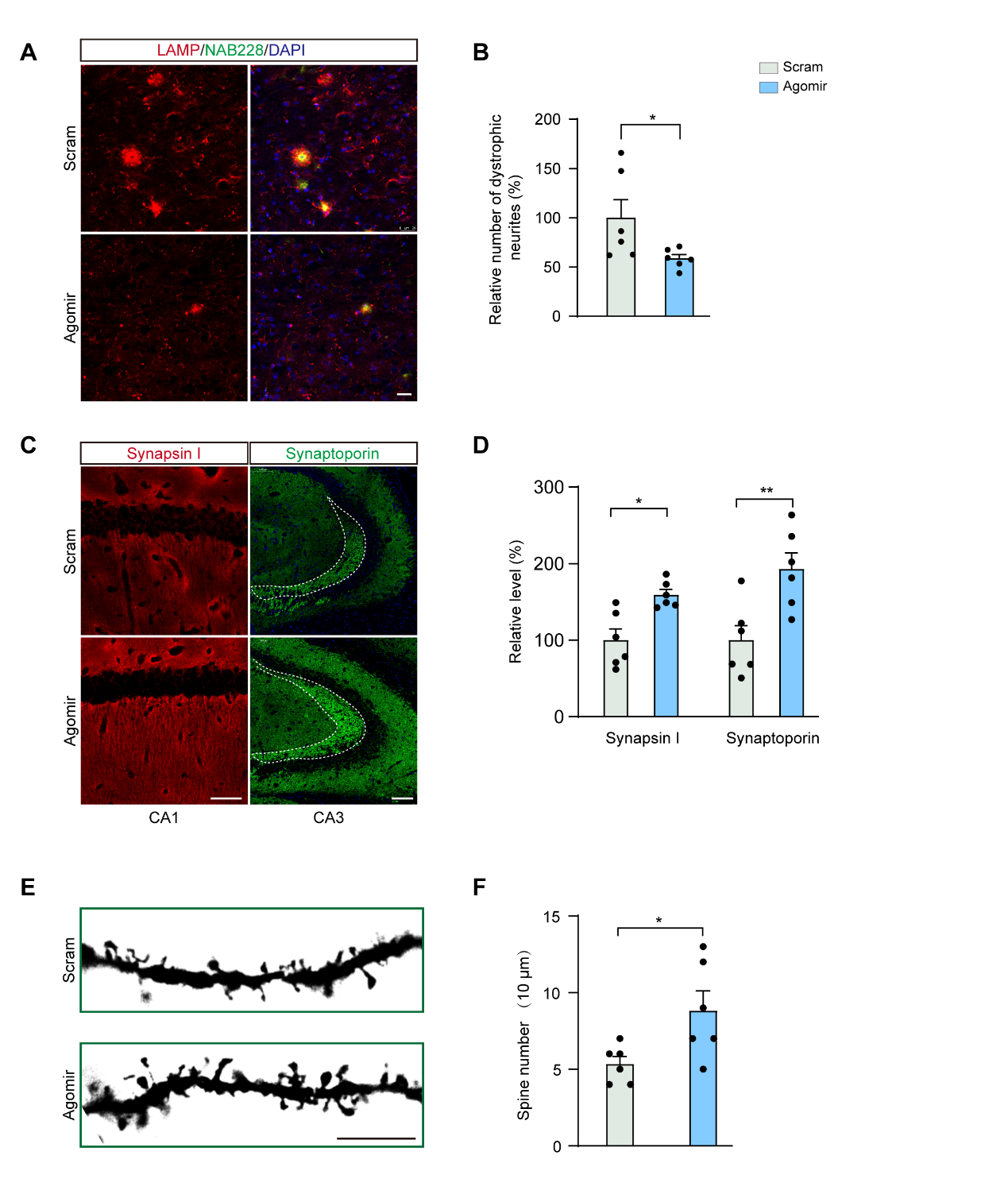


**Figure S7 AgomiR-425 oligonucleotide treatment ameliorates neuritic and synaptic pathologies.**

**(A, B)** Immunofluorescence and quantification of dystrophic neurites around the amyloid plaques in AgomiR-425 oligonucleotide and scramble treatment APP/PS1 mice. Scale bars, 25 μm. **(C, D)** Immunofluorescence and quantification of synapse-associated proteins Synapain I and synaptoporin in AgomiR-425 oligonucleotide and scramble treatment APP/PS1 mice. Scale bars, 50 μm. **(E, F)** Golgi-staining and dendritic spines quantification in AgomiR-425 oligonucleotide and scramble treatment APP/PS1 mice. Scale bars, 8 μm. Data were presented as Mean ± SEM. Two-tailed unpaired student’s t test. ***p < 0.001; *p < 0.05.


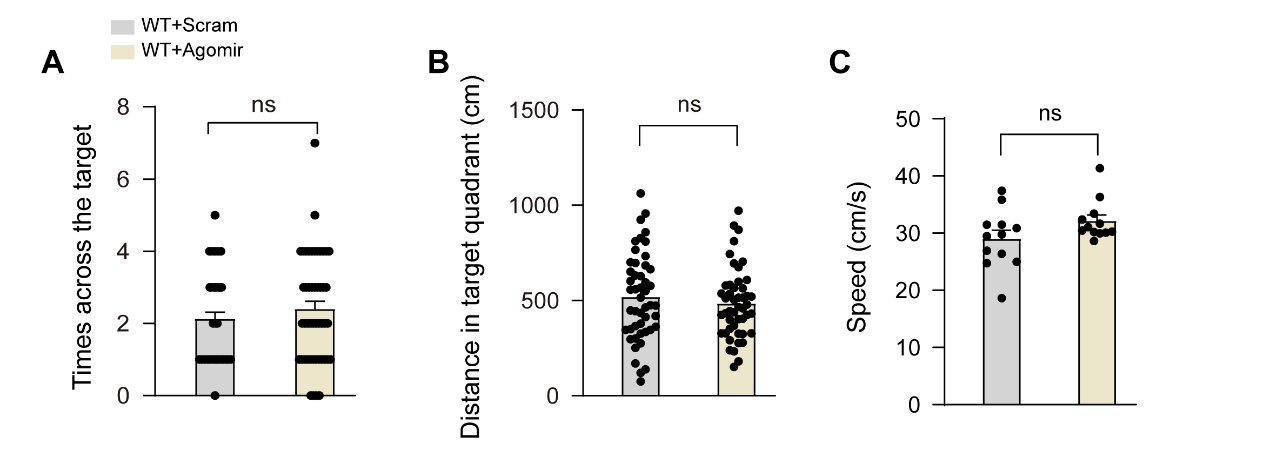


**Figure S8 AgomiR-425 oligonucleotide treatment on WT mice**

MWM data showing times across the target, distance in target quadrant for four times and swimming speed of WT mice, n=12 per group.

Data were presented as Mean ± SEM. Two-tailed unpaired student’s t test. ***p<0.001; *p<0.05.


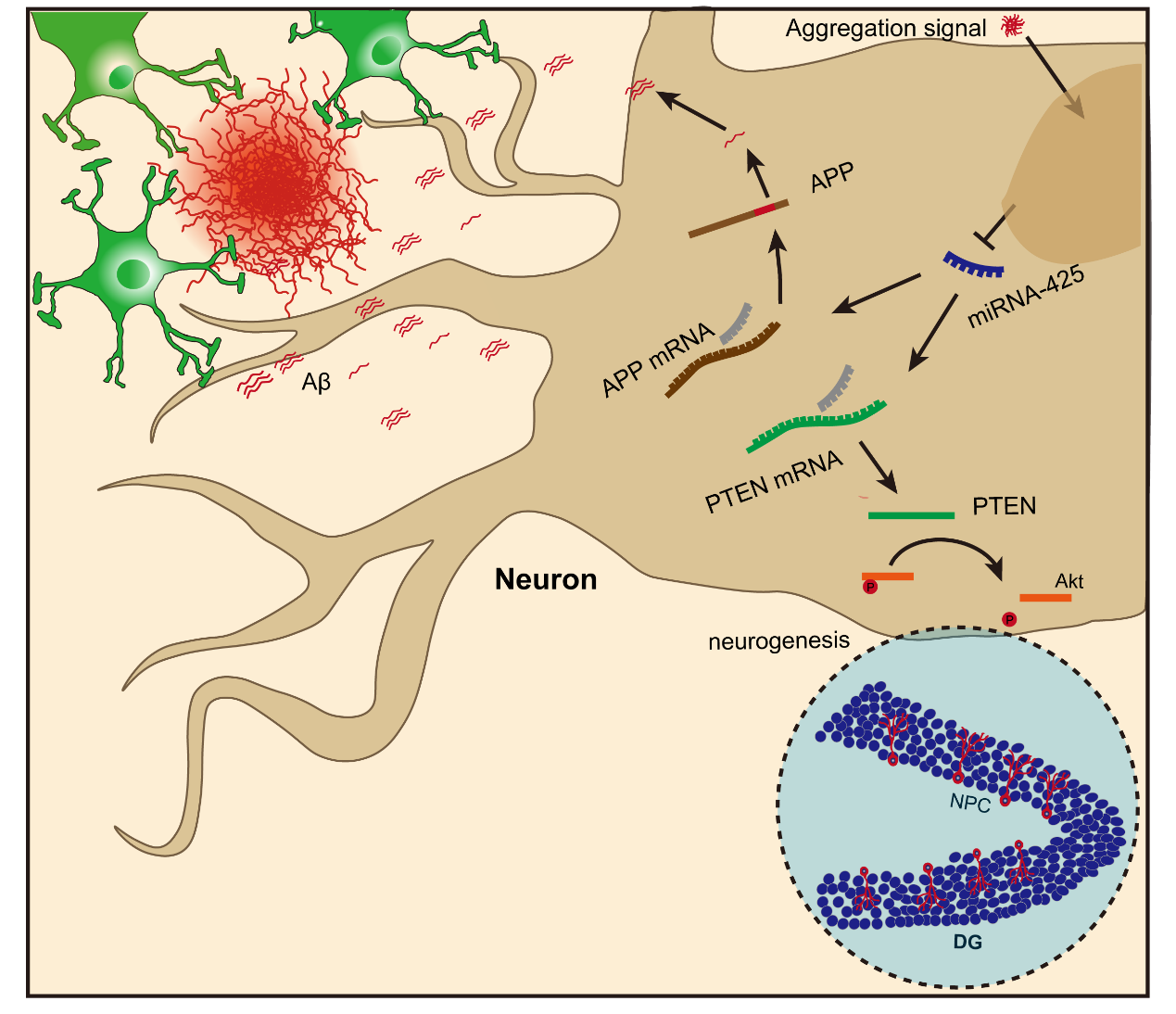


**Figure S9 Schematic model of miR-425 loss mediating APAM changes in the brain of AD.** The present study demonstrate that miR-425 loss is responsible for PI3K-Akt signaling suppression and impaired NPC proliferation. Moreover, miR-425 loss induces dendritic spine defects and impaired synaptic plasticity. Taken together, miR-425 loss following initial plaque deposition is the opening of the floodgates that mediates the ability of the APAM to cascade into a cellular phase of AD.

**Supplementary tables**

**Table S1 Detailed information for human brain samples**

**
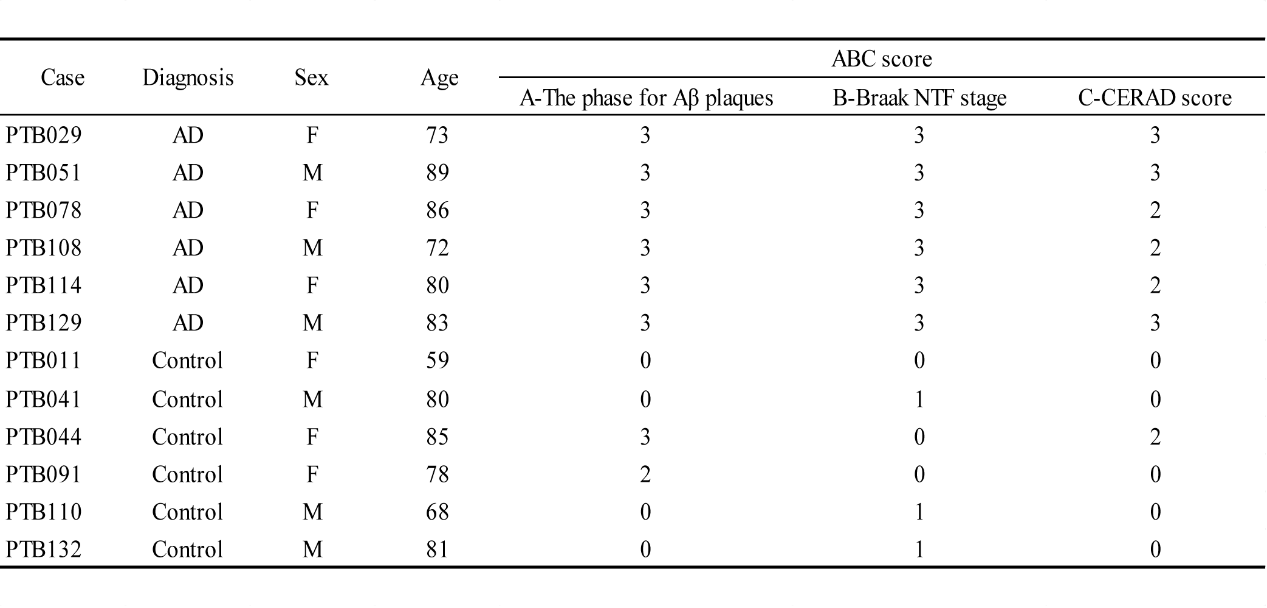
**

**Table S2 Primer sequences for RT-PCR**


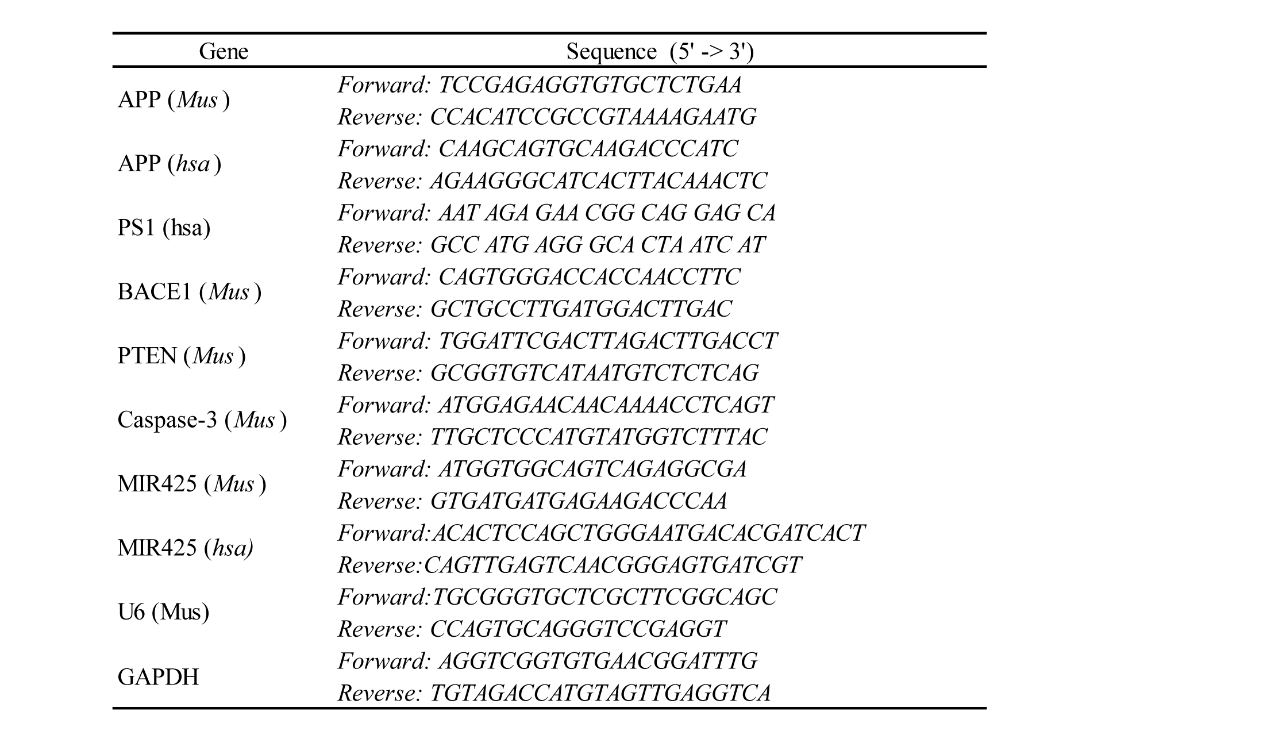

Supplement: Supplementary file 1 — Supplementary Material [file ACEL-20-e13454-s001.doc]
